# Microtubule association of TRIM3 revealed by differential extraction proteomics

**DOI:** 10.1242/jcs.261522

**Published:** 2024-01-31

**Authors:** Hannah L. Glover, Marta Mendes, Joana Gomes-Neto, Emma V. Rusilowicz-Jones, Daniel J. Rigden, Gunnar Dittmar, Sylvie Urbé, Michael J. Clague

**Affiliations:** ^1^ Department of Biochemistry, Cell and Systems Biology, ISMIB, University of Liverpool, Liverpool L69 3BX, UK; ^2^ Proteomics of Cellular Signalling, Department of Infection and Immunity, Luxembourg Institute of Health, L-1445 Strassen, Luxembourg; ^3^ Department of Life Sciences and Medicine, University of Luxembourg, 2 Avenue de l'Université, Campus Belval, L-4365 Esch-sur-Alzette, Luxembourg

**Keywords:** Microtubules, Proteomics, Nocodazole, Taxol, TRIM, LGALSL

## Abstract

The microtubule network is formed from polymerised tubulin subunits and associating proteins, which govern microtubule dynamics and a diverse array of functions. To identify novel microtubule-binding proteins, we have developed an unbiased biochemical assay, which relies on the selective extraction of cytosolic proteins from U2OS cells, while leaving behind the microtubule network. Candidate proteins are linked to microtubules by their sensitivities to the depolymerising drug nocodazole or the microtubule-stabilising drug taxol, which is quantitated by mass spectrometry. Our approach is benchmarked by co-segregation of tubulin and previously established microtubule-binding proteins. We then identify several novel candidate microtubule-binding proteins, from which we have selected the ubiquitin E3 ligase tripartite motif-containing protein 3 (TRIM3) for further characterisation. We map TRIM3 microtubule binding to its C-terminal NHL-repeat region. We show that TRIM3 is required for the accumulation of acetylated tubulin, following treatment with taxol. Furthermore, loss of TRIM3 partially recapitulates the reduction in nocodazole-resistant microtubules characteristic of α-tubulin acetyltransferase 1 (ATAT1) depletion. These results can be explained by a decrease in ATAT1 following depletion of TRIM3 that is independent of transcription.

## Teaser

**Summary:** A new method of cytosolic extraction with varying MT status is used to identify novel microtubule-associated proteins from proteomic analysis. Among these is the ubiquitin E3 ligase TRIM3.

## INTRODUCTION

Microtubules (MTs) are formed from α- and β-tubulin heterodimer subunits, which polymerise to form hollow fibres, making up one of three major cytoskeletal elements (Wickstead and Gull, 2011). They are polar structures, defined by a growing plus-end oriented towards the cell periphery and a minus-end which is usually stabilised at the microtubule-organising centre (MTOC) (Mitchison, 1993). MT networks are able to grow and shrink via continuous rounds of polymerisation and depolymerisation, allowing for constant cytoskeleton remodelling derived from their dynamic instability (Mitchison and Kirschner, 1984). Consequently, they contribute to a wide variety of cellular functions, such as maintaining cell shape, cell movement and cell division, and provide the main structural unit for flagella and cilia (Nogales, 2000). They also provide tracks along which motor proteins can travel to distribute their cargo such as organelles and membranous vesicles (Barlan and Gelfand, 2017; Bloom and Endow, 1995).

Numerous microtubule-associated proteins (MAPs) account for this diverse array of functions. Some specifically bind to the end of MTs to either control their attachment to cellular structures (−end) or their dynamics (+end). The first MAPs were discovered during the development of protocols for tubulin purification (Sloboda et al., 1975; Souphron et al., 2019). Similarly, the protein Tau was identified from porcine brain extracts, as a factor governing MT polymerisation (Weingarten et al., 1975). Modern liquid chromatography-tandem mass spectrometry (LC-MS/MS) techniques provide a large-scale, global and unbiased method for identifying MAPs. Prior MAP-centric proteomic studies have been performed using a variety of species. Over 250 MAPs were identified from early *Drosophila* embryos using taxol- and GTP-stabilised endogenous tubulin preparations, followed by 2D gel electrophoresis (Hughes et al., 2008). In a separate study, macrophage extracts were incubated with the MT-stabilising drug taxol and purified bovine brain tubulin, with candidate MAPs being identified by spectral counting (Patel et al., 2009). In 2010, more than 300 proteins from meiotic *Xenopus* egg extracts were found to bind to taxol-stabilised bovine brain tubulin (Gache et al., 2010). A final example identified over 1000 proteins binding *in vitro* to the metaphase spindle isolated from CHO cells, using multi-dimensional protein identification technology (Bonner et al., 2011).

The majority of these studies preceded major advances in the sensitivity of mass spectrometry instruments and the adoption of isotopic labelling procedures [e.g. stable isotope labelling by amino acids in cell culture (SILAC), DML and TMT] that provide more quantitative data. Furthermore, many involve the manipulation of the MT network *in vitro* and/or the addition of exogenous tubulin, thereby incompletely capturing the intracellular architecture and environment. In this study, we take a different approach, by identifying the proteins that remain bound to a residual MT network, assembled in U2OS cells under physiological conditions, following the extraction of cytosol. Our principal criterion for presuming MT association is a sensitivity to the depolymerising drug nocodazole. We describe the development of this methodology and the general features of our results. Our analysis revealed several novel candidate MT-binding proteins including the ubiquitin E3-ligase tripartite motif-containing protein 3 (TRIM3), for which we present a detailed characterisation of its association and describe effects on MT properties.

## RESULTS

Nocodazole interferes with MT polymerisation, leading to a rapid loss of the MT network upon application to cells in culture (De Brabander et al., 1977). Conversely, taxol induces the assembly and stabilisation of MTs (De Brabander et al., 1981). Following extraction of the cytosol from cells, leaving behind the MT network, proteins which show sensitivity to either of these drugs become strong candidates for MT association. By labelling the proteins in each condition with amino acids bearing different stable isotopes, residual non-MT related (or drug insensitive) proteins are simply discounted by virtue of their 1:1 ratios, which are derived from subsequent mass spectrometry analysis. We set out to determine this form of MAPome, which in contrast to previous approaches, reflects the MT status in living cells.

### Optimisation of the MT extraction protocol

**Fig. 1.**
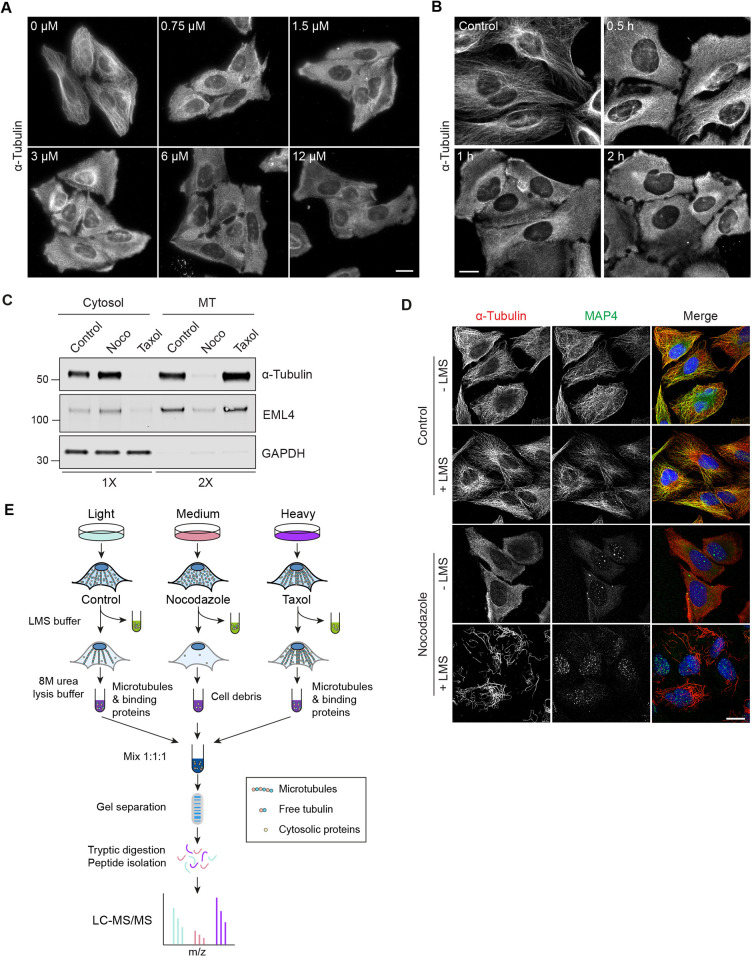
**Establishing the MAPome identification workflow.** (A) U2OS cells were treated with the indicated concentrations of nocodazole at 37°C for 30 min. Cells were fixed with methanol and stained for α-tubulin. Scale bar: 20 µm. Images were obtained using a Nikon Ti Eclipse microscope, with a CFI Plan Apo 40× objective. (B) U2OS cells were treated with 6 μM nocodazole for 0.5, 1 or 2 h. Cells were fixed with methanol and stained for α-tubulin. Scale bar: 10 μm. Images were obtained using a Nikon Ti Eclipse microscope, with a CFI Plan Apo 60× objective. (C) U2OS cells were treated with vehicle (DMSO), nocodazole (6 µM, 1 h) or taxol (6 µM, 30 min). Lysis and microtubule stabilisation buffer (LMS) was added (5 min, 4°C) to remove cytosolic proteins and residual cellular material (MT; microtubules) was then collected following 8 M urea lysis buffer extraction. Samples were analysed by western blot as indicated, loading twice as much MT fraction as cytosol). Blots are representative of three independent experimental repeats. (D) U2OS cells were treated with vehicle (DMSO) or nocodazole for 1 h. Cells were either fixed immediately or first treated with LMS to extract cytosolic proteins prior to fixation in methanol and staining for α-tubulin (red), MAP4 (green) and DAPI (blue). Scale bar: 20 µm. Images were obtained using a 3i spinning disc confocal microscope with a Plan-Apochromat 63×/1.4NA Oil Objective M27. The images shown in A, B and D are representative of each concentration, time point or condition. (E) Schematic showing the workflow for SILAC MS analysis. U2OS cells labelled with light (Lys0, Arg0), medium (Lys4, Arg6) or heavy (Lys8, Arg10) amino acids were treated with either DMSO alone (control), nocodazole or taxol, respectively, as described in C. Cells are then treated with LMS buffer at 4°C for 5 min to remove cytosolic proteins and leave behind MTs and their associated proteins. 8 M urea lysis buffer is then added to collect the residual cellular material including MTs. These fractions are mixed at a 1:1:1 ratio and analysed by MS.

Our initial approach involved an iteration of a previously described method to extract MT-associated proteins directly from U2OS cells (Duerr et al., 1981). A buffer that lyses cells, while maintaining MTs (lysis and microtubule stabilisation buffer, LMS), is added to remove cytosolic proteins and small molecules, including any free tubulin present. Vehicle-treated cells are depleted of cytosol, whereas the intact MTs and MAPs are retained. These can then be visualised by immunofluorescence microscopy or immunoblotting. For comparison, we used a condition pre-treated with the MT-depolymerising drug nocodazole, which provides us with a MT-depleted residual fraction. We first optimised the nocodazole incubation conditions required to achieve maximum MT depolymerisation. U2OS cells were treated with increasing concentrations of up to 12 µM for various times at 37°C (Fig. 1A,B). On this basis we selected a concentration of 6 µM for 1 h as the optimal treatment, although a minor fraction of MTs remained resistant to depolymerisation. Rather than use high Ca^2+^ conditions to release MAPs after cytosol extraction as described by Duerr et al. (1981), we simply recovered the cell remnants (with and without prior nocodazole extraction) with 8 M urea lysis buffer. Prior to a large scale mass spectrometry analysis, we checked that the established MAPs EML4 and MAP4 demonstrated the expected behaviour by western blotting and immunofluorescence, respectively (Fig. 1C,D). A schematic of the fully optimised protocol is shown in Fig. 1E.

### Determination of the MT proteome

**Fig. 2.**
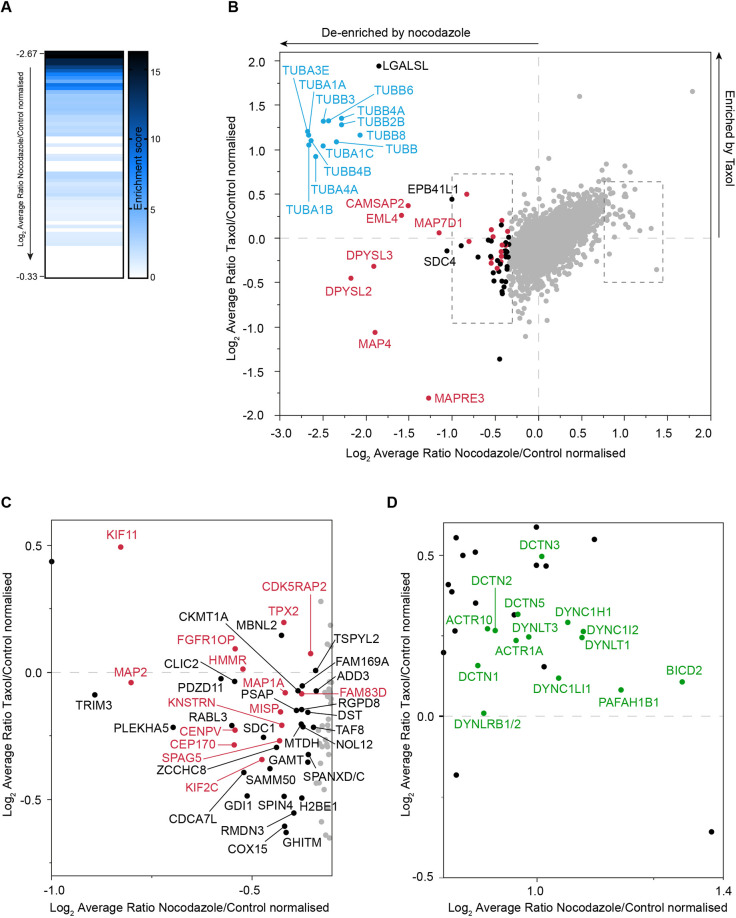
**Global overview of nocodazole and taxol sensitivity.** (A) Heat map showing the enrichment score of MT proteins. Proteins ranked according to their degree of de-enrichment after nocodazole treatment (i.e. most negative *x*-axis value) were analysed using the functional annotation clustering tool in DAVID 6.8 (Huang da et al., 2009). Consecutive groups starting with proteins ranked 1–10 followed by hits 2–11 and so on, were assessed for their enrichment score for MT association. This analysis was performed in iterative fashion until no enrichment was evident for >10 consecutive groups. (B) The log_2_ average normalised ratio of all extracted proteins in control (DMSO-only treated; light) cells compared to nocodazole (medium, x-axis) and taxol (heavy, y-axis) treatments. The proteins that are de-enriched in the nocodazole fraction were defined using DAVID as in A. Tubulin proteins are in blue, known MT-binding proteins in red and other identified binding proteins in black. (C,D) Expanded views of proteins enriched following nocodazole treatment with known MT-binding proteins shown in red (C) or green (D). All data shown in the figure are collated from three independent experimental repeats.

To determine the MT associated proteome via quantitative mass spectrometry, we generated extracts from SILAC labelled U2OS cells subjected to three different scenarios, untreated control or prior treatment with nocodazole and taxol, respectively (Fig. 1E). The three residual fractions were combined in a 1:1:1 ratio and analysed by mass spectrometry, for which full results are summarised in Table S1. The log_2_ transformed ratio of nocodazole-to-control values were ranked from the highest negative score to the lowest. To confirm MT protein enrichment and define a cut-off point for further consideration, hits were analysed in sequential groups of 10 (1–10, 2–11 etc.) for their enrichment score for ‘MT/MT-binding/MT cytoskeleton’ using DAVID Bioinformatics Resource 6.8 until no enrichment was observed for 10 consecutive groups (Fig. 2A) (Huang da et al., 2009). In effect this gives us an inferred Bayesian approach to determine a cut-off value for selecting MAP candidates from our data, for which data points of interest are indicated in Fig. 2B. The distribution of the normalised nocodazole-to-control ratio (*x*-axis) versus the normalised taxol-to-control ratio (*y*-axis) is shown in Fig. 2B–D. Proteins represented by data points in the top left quadrant of this graph will have been lost due to nocodazole treatment and preferentially retained following taxol treatment. The plotted ratios derive from averaging across the identified constituent peptides of any given protein. The behaviour of each individual peptide from selected proteins is represented in Fig. S1A. Fig. S1B shows a heat map of outlying nocodazole sensitive proteins across three independent repeats.

As expected, the strongest outliers in terms of nocodazole sensitivity are 12 isotypes of tubulin (labelled in blue, Fig. 2B). These are also modestly enriched by taxol treatment. We were surprised to find galectin-related protein (LGALSL), to be so strongly aligned with this cluster. This small protein is a candidate amyotrophic lateral sclerosis (ALS) gene, which contains a galectin domain, although most of the residues critical for carbohydrate binding are not conserved (Gelfman et al., 2019). We established that LGALSL is a bona fide MAP through immunofluorescence visualisation. Although the majority of the protein is cytosolic, concentration at MTs can clearly be discerned (Fig. S2A, top row, inset). This becomes even more obvious during cytokinesis, where MTs are bundled either side of the midbody (Fig. S2B). Although masked by the large cytosolic pool in control cells, the MT association becomes more discernible upon cytosol extraction with LMS and is disrupted by nocodazole treatment (Fig. S2A, second and third rows). Other nocodazole-sensitive outliers include echinoderm MT-associated protein-like 4 (EML4), and the tightly clustered dihydropyriminidase like 2 and 3 (DPYSL2 and DPYSL3), also known as collapsin response mediator protein-2 and -4, respectively (Fig. 2B). DPYSL2 and 3 can form heterotetramers that have been shown to enable MT growth (Fukata et al., 2002; Tan et al., 2015). MT minus-ends that are not attached to the centrosome are frequently tethered to the Golgi and can be stabilised by calmodulin-regulated spectrin-associated 2 (CAMSAP2; sensitive to nocodazole only, Fig. 2B) (Hendershott and Vale, 2014). Amongst these proteins de-enriched by nocodazole treatment and clearly separated from the main cloud of data-points, we find the ubiquitin E3 ligase family member tripartite motif containing 3 (TRIM3), for which we provide a detailed characterisation below (Fig. 2C).

Within the bottom left quadrant of Fig. 2B, we identify proteins that are lost upon both nocodazole and taxol treatments (i.e. those that associate with dynamically unstable MTs). Prominent here is MAP4, which is displaced by taxol treatment (Xiao et al., 2012), but also microtubule associated protein RP/EB family member 3 (MAPRE3), more commonly known as EB3. It is a plus-end-tracking protein (+TIP) that binds to the plus-end of MTs and regulates the dynamics of the MT cytoskeleton.

We were surprised to find a coherent group of proteins separated from the cloud on the right-hand side of the X-axis i.e. enriched by nocodazole treatment (labelled in green in Fig. 2D). This group contains multiple subunits of the cytoplasmic dynein and dynactin complexes: the minus-end directed MT motor protein and its essential cofactor respectively (Trokter et al., 2012; Urnavicius et al., 2015). Bicaudal D2 (BICD2) is an adaptor protein, which recruits dynein and stabilises the interaction between dynein and dynactin. Platelet-activating factor acetylhydrolase 1b regulatory subunit 1 (PAFAH1B1, also known as LIS1), is required for dynein-mediated transport and accumulation of dynein at MT plus-ends (Splinter et al., 2012). Finally, actin-related protein 10 (ACTR10) has been shown to induce dynein and dynactin interaction (Zhang et al., 2008). Confirmatory results by western blotting for cytoplasmic dynein 1 light intermediate chain 1 (DYNC1LI1) are shown in Fig. S2C. Immunofluorescence microscopy reveals a translocation of DYNC1LI1 to the nuclear membrane upon nocodazole treatment, which is retained upon LMS extraction (Fig. S2D).

### TRIM3 – a novel MT-binding protein

**Fig. 3.**
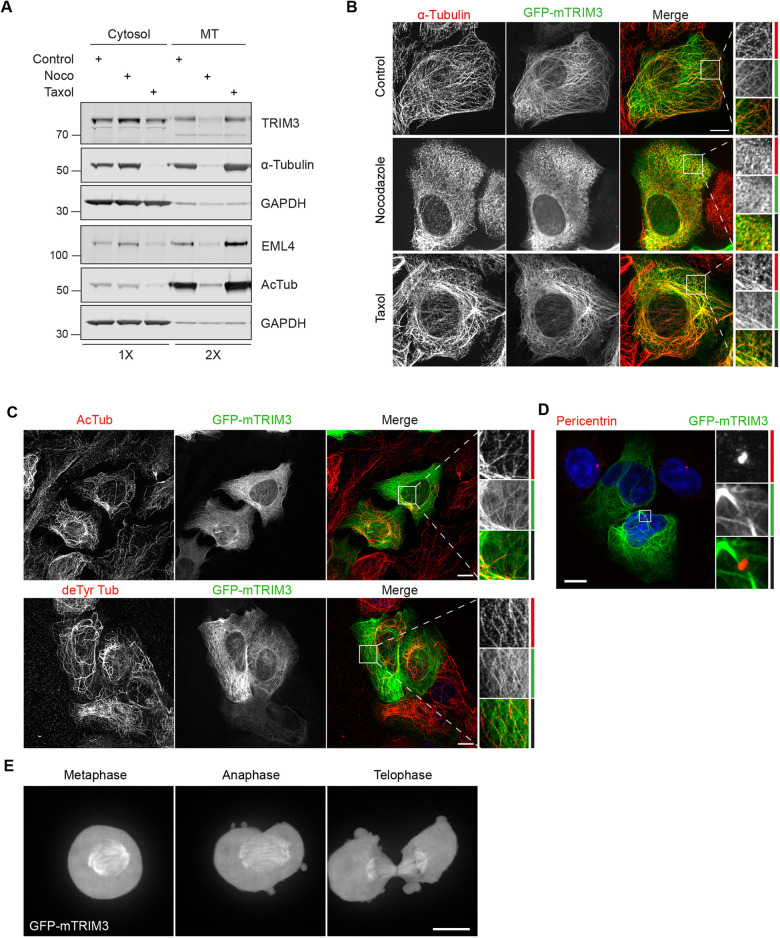
**Validation and characterisation of TRIM3 MT binding.** (A) U2OS cells were treated with either nocodazole (6 μM, 1 h) or taxol (6 μM, 30 min) alongside a DMSO-only treated control sample. LMS buffer was then added (5 min, 4°C) to remove cytosolic proteins (Cytosol). Residual cellular material, including MTs, was then collected using 8 M urea lysis buffer. Samples were analysed by western blotting as indicated, loading twice as much MT fraction as cytosol. (B) GFP–mTRIM3-transfected U2OS cells were treated with DMSO (1 h, Control), nocodazole (6 µM, 1 h) or taxol (6 µM, 30 min) before fixation and staining for α-tubulin (red). (C) GFP–mTRIM3 transfected cells were fixed and stained for either acetylated tubulin (AcTub) or detyrosinated tubulin (deTyr Tub) (red). (D) GFP–mTRIM3-transfected cells were fixed and stained for pericentrin (red) and with DAPI (blue). (E) GFP–mTRIM3-transfected cells were synchronised using thymidine and nocodazole to arrest cells at prometaphase. Nocodazole was washed out and *z*-stacks were acquired every minute (range 15 µm, step 1 µm) as cells progressed through mitosis. A maximum projection is shown. All immunofluorescence experiments were fixed in ice-cold methanol and imaged using a 3i spinning disc confocal microscope, with Plan-Apochromat 63×/1.4 NA oil objective M27. Scale bars: 10 µm. All data shown in the figure are representative of three independent experimental repeats.

As detailed above, TRIM3, also known as BERP, is clearly identified as an outlier protein that is de-enriched by nocodazole treatment. This sensitivity was confirmed by immunoblots showing the corresponding extraction pattern (Fig. 3A). It belongs to the TRIM family of E3 ligases, some of which have been previously localised to MTs (Meroni, 2012). So far, MT association has been confined to TRIM proteins that possess a COS motif, FNIII and SPRY/B30.2 domain as their C-terminal domain arrangement (Cox, 2012; Short and Cox, 2006). To determine whether TRIM3 is truly a novel MAP, mouse GFP–TRIM3 was transiently transfected into U2OS cells and cells co-stained for α-tubulin, with which it showed a clear colocalisation (Fig. 3B). This typical distribution was lost upon nocodazole treatment but remained evident with taxol. Thus, we have been able to use our proteomics approach to identify a second novel MAP alongside LGALSL reported above.

MTs are highly decorated with a large number of post-translational modifications (PTMs), and it has come to light that these modifications can play a role in directing which MAPs can associate with which MT subsets (Janke and Magiera, 2020). Therefore, we analysed whether TRIM3 colocalises with a particular MT subset. Fig. 3C illustrates that GFP–TRIM3 is not excluded from, nor specifically associated with, either detyrosinated or acetylated MTs. However, some of the more intense, seemingly bundled, detyrosinated MT structures were devoid of TRIM3. Furthermore, TRIM3 did not associate with the centrosome (Fig. 3D). The MT network undergoes major redistribution during mitosis. Following the behaviour of TRIM3 through mitosis in synchronised cells, we observed GFP–TRIM3 on the mitotic spindle from metaphase right through to telophase at which point it equally decorated the central spindle MTs (Fig. 3E; Movie 1). The closest paralogue to TRIM3 is TRIM2, and GFP–TRIM2 also shows a clear MT association, which has not hitherto been reported (Fig. S3A). Like TRIM3, GFP–TRIM2 also associated with a subset of acetylated and detyrosinated MTs but is absent from the centrosome (Fig. S3B,C).

### The C-terminal region is responsible for TRIM3 localisation to MTs

**Fig. 4.**
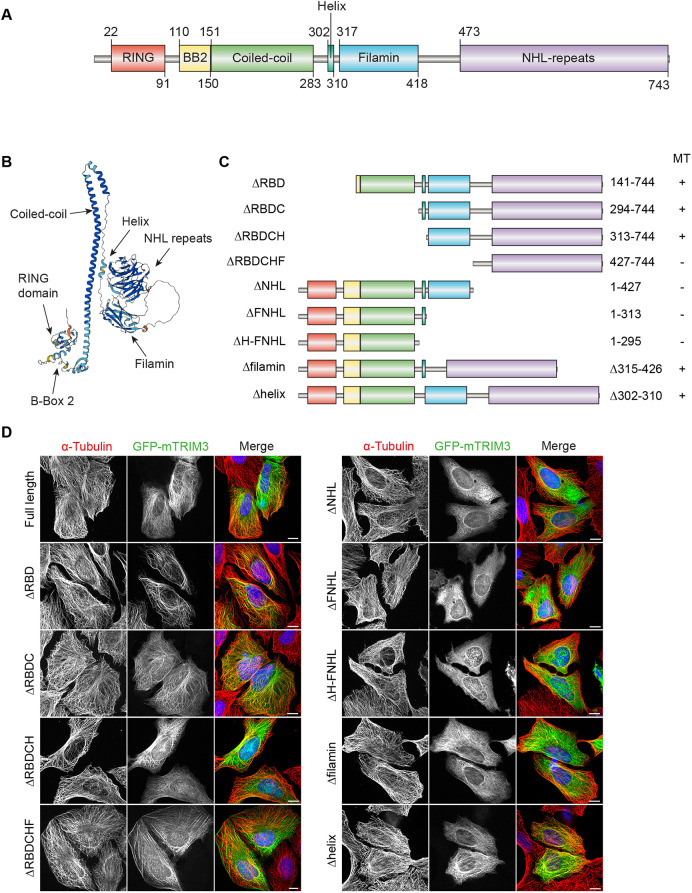
**TRIM3 associates with MTs via its C-terminus.** (A) Schematic representation of full-length of TRIM3 and its domains. (B) 3D structural prediction of TRIM3 with domains indicated. Obtained from AlphaFold database, AF-O75382-F1. (C) Schematic representation of the TRIM3 deletion constructs used to map the MT-localising region. MT-binding capability is indicated by + and −. (D) Representative images of TRIM3 deletion constructs co-stained with α-tubulin. Images were acquired using a 3i spinning disc confocal microscope with Plan-Apochromat 63×/1.4 NA oil objective M27. Scale bars: 10 µm. Data in D are representative of two independent experimental repeats.

TRIM family proteins are defined by their common domains at their N-terminal end and are divided into subfamilies based on their C-terminal domains. Inspection of the 3D structures of TRIM proteins predicted by AlphaFold2 revealed that all COS-box proteins as well as TRIM3 contain a short α-helix structure shortly after the coiled-coil domain. A schematic and AlphaFold2-derived structural model of TRIM3 are shown in Fig. 4A and B, respectively, illustrating the family specific tripartite motif (RING, BB2, coiled-coil) followed by the short α-helix, a filamin domain and NHL repeats. Sequence alignments of human TRIM family members reveal that the conserved amino acids within the COS-box, which have been shown to be required for MT localisation in other family members, are not present within TRIM3 or TRIM2 (Fig. S4; Short and Cox, 2006). This suggests that MT network localisation is achieved by other means. In order to map which region of TRIM3 is required for MT localisation, nine different GFP-tagged truncation or deletion constructs were generated (Fig. 4C). Removal of the tripartite motif (ΔRBDC) did not affect MT localisation, as a truncation retaining the helix, filamin and NHL repeats in isolation was still able to successfully colocalise with tubulin (Fig. 4C,D). Concordantly, the N-terminal tripartite motif-only construct (ΔH-FNHL) did not bind MTs and the C-terminal region is therefore solely responsible for MT binding. Further analysis of the constructs implicates the C-terminal NHL-repeat region in conjunction with the filamin domain as the minimal determinant of MT patterning. Interestingly, the NHL domain alone (ΔRBDCHF) appears to specifically relocate to actin stress fibres.

### Taxol and nocodazole treatments reveal an influence of TRIM3 on MT properties

**Fig. 5.**
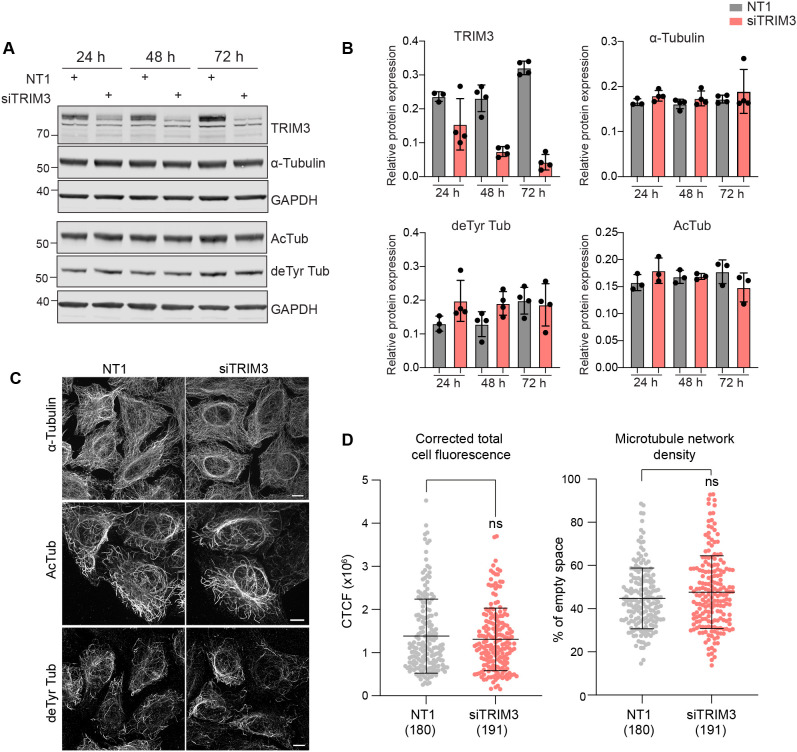
**TRIM3 depletion does not affect the global MT network.** (A) U2OS cells were transfected with control (NT1) or TRIM3 siRNA (siTRIM3) for 24, 48 or 72 h prior to analysis by western blotting. (B) Quantification of relative expression levels showing mean±s.d. for three or more independent biological experiments for each condition. (C) U2OS cells were transfected with control (NT1) or TRIM3 targeted siRNA (siTRIM3) for 48 h. Cells were fixed with ice-cold MeOH and stained for α-tubulin, acetylated tubulin (AcTub) or detyrosinated tubulin (deTyr Tub). Images were acquired using a 3i spinning disc confocal microscope with a Plan-Apochromat 63×/1.4NA Oil Objective M27. Scale bar: 10 µm. (D) Quantitative assessment of the MT network showing the corrected total cell fluorescence (CTCF, left) and the percentage of empty space (right) from three independent experiments. Mean±s.d. shown for the indicated number of cells. Statistical analysis (ns, not significant) was determined using a two-tailed unpaired Student's *t*-test.

**Fig. 6.**
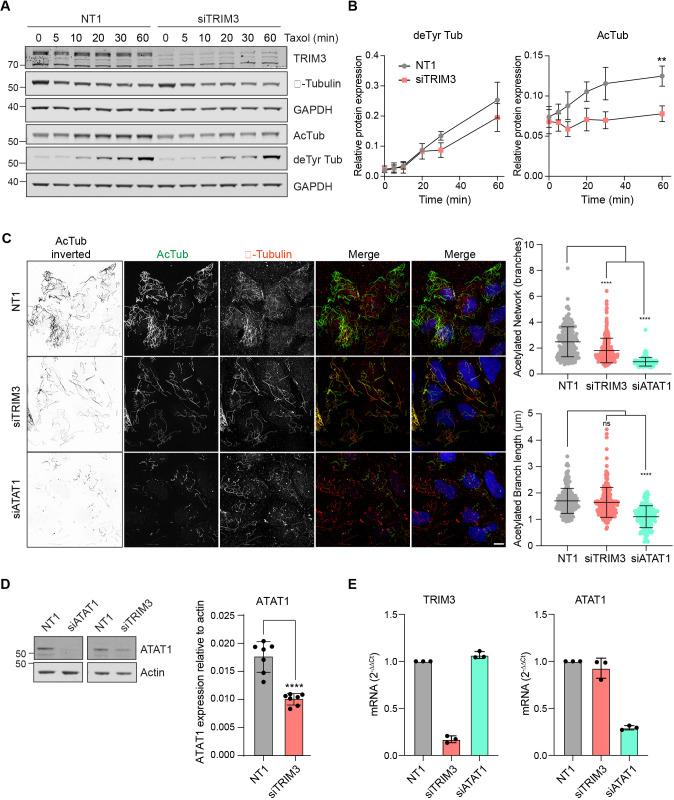
**TRIM3 enables drug-sensitive acetylation of MTs.** (A) U2OS cells were transfected with non-targeting control (NT1) or TRIM3 targeting siRNA (siTRIM3) for 72 h. Transfected cells were treated with 0.5 µg/ml taxol for indicated times and expression levels of tubulin and modified tubulin were analysed. (B) Quantification of detyrosinated (deTyr) and acetylated tubulin (AcTub) levels for results as in A. Mean±s.d. of three (deTyr) and four (AcTub) independent experiments are shown. **P≤0.01 (two-way ANOVA with Šídák's multiple comparisons test). (C) U2OS cells were transfected with non-targeting control (NT1) or siRNA targeting TRIM3 or ATAT1 for 72 h. Transfected cells were treated with 6 µM nocodazole for 1 h. Cells were then washed in PHEM buffer to remove the depolymerised MTs, leaving behind only stable MTs. Cells were fixed with ice cold MeOH and stained for β-tubulin (red) and acetylated tubulin (AcTub, green). Images were acquired using a 3i spinning disc confocal microscope with a Plan-Apochromat 63×/1.4NA Oil Objective M27. Scale bar: 10 µm. Quantification using MiNA analysis shows the mean number of branches per network (mean branch network) and branch length of acetylated MTs. Cell numbers analysed: siNT1 (189), siTRIM3 (217), siATAT1 (161). Mean±s.d. are shown. ****P≤0.0001; ns, not significant (one-way ANOVA with Tukey's multiple comparison test). (D) U2OS cells were transfected with control (NT1) or siRNA targeting TRIM3 or ATAT1 for 72 h. Cells were lysed in RIPA buffer and ATAT1 expression levels were analysed by western blotting. Graph shows quantification of ATAT1 expression levels relative to actin from seven independent experiments. Mean±s.d. are shown. ****P≤0.0001 (two-tailed unpaired *t*-test). (E) U2OS cells were transfected with control or siRNA targeting either TRIM3 of ATAT1 for 72 h. TRIM3 and ATAT1 mRNA levels relative to GAPDH were determined using qRT-PCR. Mean±s.d. are shown.

We next sought to define a role for TRIM3 in relation to MT network properties. TRIM3 was efficiently depleted using siRNA transfection, and immunoblotting and immunofluorescence analyses were performed. Data presented in Fig. 5A,B, confirms that levels of total, acetylated or detyrosinated tubulin were unchanged following depletion for all indicated time frames. Visual inspection and quantitative analysis did not reveal any obvious perturbation of the MT network (Fig. 5C,D; Fig. S5A). MT networks that are stabilised with acute taxol treatment undergo an increase in detyrosination and acetylation. To determine whether TRIM3 plays a role in the accumulation rate of these MT modifications, TRIM3 was depleted for 72 h before treating with taxol over a 60 min period. Quantification from three independent repeats revealed that the accumulation of detyrosination was not significantly affected; however, acetylation was unable to further accumulate under these conditions despite starting from identical baseline levels (Fig. 6A,B). Acetylation is a marker of long-lived MTs and has previously been shown to be involved in protecting MTs from mechanical ageing (Portran et al., 2017; Xu et al., 2017). Depletion of the enzyme responsible for MT acetylation, alpha-tubulin acetyltransferase 1 (ATAT1) leads to changes in the nocodazole-resistant fraction of MTs, a finding we have reproduced here (Fig. 6C, bottom panel; Xu et al., 2017). Correspondingly, we find that TRIM3 depletion partially recapitulates this phenotype, wherein the levels of residual acetylated MTs are clearly sparser. This influence of TRIM3 can be accounted for by a 50% reduction in ATAT1 levels upon TRIM3 depletion, which is not due to effects upon transcription, as mRNA levels are unchanged (Fig. 6D,E). Although this provides a plausible mechanistic link for the impact of TRIM3 depletion on MT acetylation, how it is effected is currently unclear. ATAT1 is a relatively stable protein (Fig. S5B,C) and any direct TRIM3 enzyme–substrate relationship would be expected to result in an increase in ATAT1 levels upon TRIM3 depletion, rather than the observed decrease.

## DISCUSSION

Our study provides two novel elements to the analysis of the ‘MAPome’. Firstly, our use of stable isotopes to conduct a triplexed experiment provides a quantitative assessment of enrichment. Secondly, it reflects interactions that occur on MT networks in the living cell rather than *in vitro*. However, as with any method that requires a physical separation of bound and unbound proteins, it will be affected by loss of low-affinity proteins. Our strategy of differential extraction makes use of the leakage of free proteins versus MT-associated proteins from the permeabilised cells. This concept has rarely been applied to quantitative proteomic studies, but we recall that differential extraction of membrane proteins by detergents played a key role in establishing the concept of ‘lipid rafts’ and in the identification of caveolin 1 (VIP21) (Kurzchalia et al., 1992).

In our study, we are also defining sets of proteins that show the highest sensitivity to two widely used drugs that act upon the MT network. This can simply reflect the relative abundance of the MT network or changed configuration of tubulin induced by drug binding. It is the nocadazole-reduced residual fraction that has been most clearly informative and represents the most novel aspect of this study. This approach is validated by the high enrichment for MT-associated proteins among the outliers, including most major tubulin isoforms (Fig. 2A,B). Our inventory provides a rich resource of uncharacterised proteins from which we have been able to show an association with MTs for two chosen examples, LGALSL and TRIM3.

Several TRIM proteins have been previously described to localise to MTs via a signature amino acid sequence, named the COS box, which follows the coiled-coil domain (Short and Cox, 2006). Mutations induced at the beginning and end of this region lead to dissociation from the MT network. TRIM3 does not possess a COS box and we have shown that the MT-localising regions lie elsewhere. We identified seven TRIM proteins within our dataset (TRIM3, TRIM24, TRIM56, TRIM28, TRIM33, TRIM23 and TRIM25), of which TRIM3 was the only outlier (Fig. S5D). We did not identify TRIM2, the closest relative of TRIM3, with which it is known to heterodimerise (Esposito et al., 2022). However, we were able to show TRIM2 MT association following exogenous expression. The most likely reason we did not find TRIM2 in our data set is its low level of expression in U2OS cells. A previous high-depth quantitative study of the whole U2OS proteome identified 23 TRIM proteins over three orders of magnitude expression levels, but failed to register TRIM2 (Beck et al., 2011).

The TRIM family have been associated with multiple cellular functions with relevance to both cancer and immunity (Hatakeyama, 2011; Ozato et al., 2008). Proposed TRIM3 substrates include toll-like receptor 3 and importin α, (Li et al., 2020; Zhu et al., 2019). It has also been shown to bind to the neuronal kinesin motor protein KIF21B, whose motility it regulates in a ligase-independent manner (Labonte et al., 2013). Our goal in this study was to develop a new approach for gaining a global view of MAPs. Accordingly, we do not provide a complete and detailed mechanistic study of TRIM3 function, but we have made some intriguing observations. Using orthologous assays, we have unveiled a subtle phenotype linking TRIM3 to the accumulation of acetylated MTs. Our finding that TRIM3 also regulates the levels of the enzyme responsible for acetylation, ATAT1, provides a plausible explanation and starting point for further investigation. We are not proposing that ATAT1 is a direct TRIM3 E3 ligase substrate leading to its degradation, as TRIM3 depletion would then result in an increase rather than the observed decrease. Additionally, TRIM3 is a relatively poor E3 ligase in isolation, but can be activated by association with its paralogue TRIM2, which, as discussed above, is not found in our data or in U2OS cells more generally (Esposito et al., 2022). At this point, we cannot rule out TRIM3-mediated effects upon ATAT1 translation, particularly as its NHL repeat domain is proposed to confer RNA-binding activity as well as the MT-binding shown here (Goyani et al., 2021).

We were surprised to find a coherent set of proteins enriched in the nocodazole-treated residual fraction. We ascribe this to the nocodazole-induced relocation of dynein-associated molecules to the nuclear membrane. Such a nocodazole-induced translocation phenomenon has been previously described for dynein, and reflects conditions in the G2 phase of the cell cycle leading to association of BICD2 and dynein with nuclear pores (Gerlitz et al., 2013; Splinter et al., 2010). This nuclear membrane redistribution has also been well described for another major outlier PAFAH1B1 (also known as LIS1) (Hebbar et al., 2008). In effect nocodazole might here be mimicking the choreography of mitosis, whereby disassembly of MTs in prophase is accompanied by dynein-dependent nuclear envelope breakdown (Salina et al., 2002).

Some proteins are at first glance counterintuitively de-enriched by both nocodazole and taxol treatments, most notably EB3 (MAPRE3). If a protein is released by nocodazole treatment, why would they also be released upon MT stabilisation? In this case, the answer would appear to lie in the specific association of EB proteins with GTP-tubulin at the growing tips of MTs, which are lost upon taxol stabilisation (Maurer et al., 2011). The dual sensitivity of EB3 in the two drug-treated conditions can be rationalised in this way and the assay is reflecting real biological behaviour. Nevertheless, the identification of MT-associated EB3 in control cells might be considered surprising given the high dissociation rate of EB family proteins (Roth et al., 2018). The related protein EB1 shows a similar trend but is below our cut-off value, perhaps reflecting a higher dissociation rate from MTs in the control condition.

In summary, we have established a new method for identification of MAPs, which we hope will translate into other cell types and prove useful in the investigation of pathological conditions. The ability to culture induced pluripotent stem cell (iPSC) neurons at scale will offer an opportunity to transfer our system to a specialised MT network (Tian et al., 2019). Our vision is that this method will be one component of a new wave of MS-based methods to comprehensively detail the MAPome. There is a broad unresolved question as to why multiple TRIM family ubiquitin E3 ligases associate with MTs, to which we now contribute two new examples. TRIM2 has been previously linked to neuronal polarisation and knockout mice show evidence of neurodegeneration (Balastik et al., 2008; Khazaei et al., 2011). TRIM2 and TRIM3 are both enriched in the brain and their functions there must be considered in the light of our findings.

## MATERIALS AND METHODS

### Cell culture

U2OS cells from the European Collection of Authenticated Cell Cultures (ECACC) were grown in Dulbecco's modified Eagle's medium (DMEM; Gibco) supplemented with 10% heat-inactivated fetal bovine serum (FBS; Gibco) (complete medium) at 37°C with 5% CO_2_. Cells were routinely tested for mycoplasma.

### Antibodies

Primary antibodies against the following proteins were used for western blotting at the indicated concentrations: α-tubulin (Sigma, T5168, 1:10,000), acetylated α-tubulin (Sigma, T6793, 1:1000), ATAT1 (Proteintech, 28828-1-AP, 1:500), detyrosinated α-tubulin (Abcam, ab32386, 1:1000), DYNC1LI1 (Atlas, HPA035013, 1:1000), EML4 (Cell Signaling Technology, 12156S, 1:1000) and GAPDH (Cell Signalling Technology, 2118S, 1:1000). Primary antibodies against the following proteins were used for immunofluorescence at the indicated concentrations: α-tubulin (Bio-Rad, MCA77G, 1:500), β-tubulin (Abcam, ab6046, 1:200), acetylated α-tubulin (Sigma, T6793, 1:1000), detyrosinated α-tubulin (Abcam, ab32386, 1:200), DYNC1LI1 (Atlas, HPA035013, 1:1000), MAP4 (Bethyl, A301-488A, 1:1000) and pericentrin (Abcam, ab4448, 1:1000). Secondary antibodies used for western blotting were obtained from LICOR Biosciences: donkey anti-mouse-IgG IRDYE 800CW (926-32212), donkey anti-mouse-IgG IRDYE 680CW (926-32222), donkey anti-rabbit-IgG IRDYE 800CW (926- 32213), donkey anti-rabbit-IgG IRDYE 680CW (926-32223). Secondary antibodies used for immunofluorescence obtained from Invitrogen were: donkey anti-rabbit-IgG Alexa Fluor (AF)488 (A21206), donkey anti-rabbit-IgG AF594 (A21207), donkey anti-mouse-IgG AF488 (A21202), donkey anti-mouse AF594 (A21203) and donkey anti-rat-IgG AF594 (A21209). Uncropped images of western blots shown in this paper can be found in Fig. S6.

### Plasmids

Full-length LGALSL was amplified by PCR from pBluescriptR-LGALSL purchased from Horizon Discovery (Cambridge, UK; MHS6278-202809144, clone ID: 5301908). Full-length TRIM2, mTRIM3 and mTRIM3 truncations and deletions were amplified by PCR from pcDNA3X(+)MycEGFP-TRIM2 and pcDNA3X(+)MycEGFP-mTRIM3 which were a kind gift from Professor Germana Meroni (University of Trieste, Italy). PCR amplicons were then subcloned into BglII and SalI sites of the pEGFP-C1 vector (Clontech).

### Drug treatments

Nocodazole was used at 330 nM for synchronisation and 6 µM for MT depolymerisation. Taxol was used at 6 µM for MT stabilisation and 600 nM for accumulation of modifications. Thymidine was used at 2 mM and cycloheximide at 100 µg/ml. The duration of treatments is detailed in respective figure legends.

### Cell lysis, SDS-PAGE and western blotting

Cells were placed on ice, rinsed twice with ice-cold PBS and lysed in RIPA buffer (150 mM NaCl, 1% Triton X-100, 0.1% SDS, 1% sodium deoxycholate and 10 mM Tris-HCl pH 7.5) supplemented with mammalian protease inhibitors (1:250 v/v; Sigma) on a rocker on ice for 15 min. All samples were suspended in SDS sample buffer (62.5 mM Tris-HCl pH 6.8, 3% SDS, 10% glycerol, 3.2% β-mercaptoethanol and 0.25% Bromophenol Blue) and boiled (95°C, 5 min). Proteins were resolved on precast NuPAGE Novex 4–12% Bis-Tris Gels in 3-(*N*-morpholino)propanesulfonic acid (MOPS) buffer (Invitrogen). Samples were transferred to 0.45 µm nitrocellulose membrane (0.9 A, 1 h) in transfer buffer (0.2 M glycine, 25 mM Tris and 20% methanol). Membranes were incubated in blocking solution [Tris-buffered saline (TBS); 150 mM NaCl, 10 mM Tris-HCl pH 7.5] supplemented with 0.1% Tween-20 (TBST, v/v; Thermo Fisher Scientific) and 5% milk (w/v; Marvel) for 1 h without antibodies and then overnight with primary antibodies, washed three times for 5 min in TBST before incubation in either IRDye680 or IRDye800-conjugated anti-mouse-IgG or anti-rabbit-IgG secondary antibodies in blocking solution for 1 h (1:15,000 v/v; LI-COR). Visualisation and quantification of western blots were performed using an Odyssey infrared scanner (LI-COR Biosciences). For western blot quantitation, raw signal values were obtained from image studio software following background subtraction. For quantification across multiple experiments, raw values were either normalised to the sum of the raw values from each individual blot (Figs 5A and 6B) or normalised to the corresponding actin bands (Fig. 6D).

### Fluorescence microscopy

Cells were seeded onto 22 mm^2^ coverslips in six-well plates. Cells were fixed with ice cold methanol (MeOH) at −20°C for 5 min, washed in PBS before incubating with 10% goat serum in PBS for 30 min at room temperature. Primary antibodies (1 h) and secondary antibodies (30 min) were applied sequentially in 5% goat serum, each followed by two 4 min washes with PBS. Coverslips were finally washed in water and mounted onto glass slides using Mowiol supplemented with DAPI. Images were acquired using either a Nikon Ti Eclipse microscope with a CFI Plan Apo 40× objective or a CFI Plan Apochromat VC 60× objective lens, or a 3i Marianas spinning disk confocal microscope (3i Intelligent Imaging innovations, Germany) with a Plan-Apochromat 40×/1.3 NA oil objective or a Plan-Apochromat 63×/1.4 NA oil objective M27 or using a Zeiss LSM900 with Airyscan confocal laser scanning microscope using a 63× 1.4 NA Zeiss Plan Apochromat objective.

### Microtubule extraction protocol

Cells were treated with 6 μM of nocodazole for 1 h or 6 μM of taxol for 30 min at 37°C alongside a DMSO control. The following steps were performed on ice using buffers pre-cooled to 4°C. Cells were washed in PBS before being incubated in lysis and microtubule stabilisation buffer [LMS; 100 mM PIPES pH 6.9, 5 mM MgCl_2_, 1 mM EGTA, 30% glycerol, 0.1% NP40, 0.1% Triton X-100, 0.1% Tween-20, 0.1% 2-mercaptoethanol (v/v)] supplemented with mammalian protease inhibitors (1:250) for 5 min before collection of lysates. The remaining MTs on the plates were then extracted using 8 M urea, 50 mM Tris-HCl pH 8 (8 M urea lysis buffer).

### siRNA transfection

All siRNA treatments were performed the day after cell seeding using Lipofectamine RNAiMAX transfection reagent, in accordance with the manufacturer's protocol. Cells were incubated for either 48 h or 72 h as specified. Sequences of siRNA oligonucleotides (all 5′ to 3′) used are as follows: ONTARGETplus Non-targeting siRNA oligo#1 (NT1) 5′-TGGTTTACATGTCGACTAA-3′ (Dharmacon, D-001810-01), TRIM3 oligo#1 GUACAGCACAGGCGGCAAA, oligo#2 GCACAUAUGAGCUAGUGUA, oligo#3 GAGCGCCACUGCACACGAA, oligo#4 GAAUGAAAUUGUAGUAACG (Dharmacon, L-006931-00), ATAT1 oligo#1 GUAGCUAGGUCCCGAUAUA, oligo#2 GAGUAUAGCUAGAUCCCUU, oligo#3 GGGAAACUCACCAGAACG and oligo#4 CUUGUGAGAUUGUCGAGAU.

### Plasmid DNA transfection

All plasmid DNA transfections were performed using 1 µg of plasmid and Genejuice or Lipofectamine 2000 the day prior to collection of experimental data in either a six-well plate or µ-Dish 35 mm high Ibidi^®^ dishes (Ibidi LCC, Martinsried, Germany) for live-cell imaging, with cells at 60–80% confluency at the time of transfection.

### Cell synchronisation and live-cell imaging

For imaging of TRIM3 on the mitotic spindle, cells were synchronised to increase the mitotic index. Cells were seeded into 35 mm ibidi dishes the day prior to synchronisation in prometaphase using 2 mM thymidine (24 h) followed by 330 nM nocodazole (16–18 h). Transfection with plasmid DNA was performed alongside nocodazole addition. To allow re-entry into mitosis, cells were quickly rinsed three times, then incubated in complete medium for 1 h prior to imaging. Cells in late prometaphase or metaphase were selected and *z*-stacks were acquired every minute as cells passed through mitosis. Imaging was acquired using a 3i Marianas spinning disk confocal microscope (3i Intelligent Imaging innovations, Germany) with a Plan-Apochromat 63×/1.4NA oil objective M27.

### Analysis of stable MTs

U2OS cells were transfected with siRNA against TRIM3 and ATAT1 alongside a non-targeting control (NT1) for 72 h. Cells were then treated with 6 µM nocodazole for 1 h to depolymerise the dynamic MTs while maintaining nocodazole-resistant ones. Cells were washed twice in a buffer containing PIPES, HEPES, EGTA and MgSO_4_ (PHEM; 60 mM PIPES, 25 mM HEPES, 10 mM EGTA and 4 mM MgSO_4_) before treatment with PHEM buffer containing 0.2% Triton-X 100 for 1 min. This removes the cytosolic tubulin (depolymerised MTs) while maintaining the intact stable MTs. Cells were then fixed and stained as described above.

### SILAC labelling

U2OS cells were cultured under standard conditions in DMEM for SILAC supplemented with 10% dialysed FBS and L-proline, Pro0 (200 μg/ml; Sigma) to prevent conversion of arginine to proline. Differentially labelled amino acids were added to the media to generate SILAC media for three conditions: light (L-lysine, Lys0; L-arginine, Arg0; Sigma), medium (L-lysine-^2^H_4_, Lys4; L-arginine-^13^C_6_, Arg6; Sigma) and heavy (L-lysine-^13^C_6_-^15^N_2_, Lys8; L-arginine-^13^C_6_-^15^N_4_, Arg10; Sigma). L-lysine was supplemented at a final concentration of 146 μg/ml whereas L-arginine was supplemented at a final concentration of 84 μg/ml. Cells were cultured for a minimum of six passages and analysed by MS to confirm amino acid incorporation before experiments proceeded.

### Mass spectrometry

Extracted MTs from each condition were combined at a 1:1:1 ratio and filtered through 15 ml Amicon ultra centrifugal filters (10,000 kDa MW cut off) at 4000 ***g*** at room temperature (RT) until the minimum volume was retained. The flow-through was discarded and the sample suspended in sample buffer before being subjected to processing for MS. SDS-PAGE was performed until proteins had migrated one-third of the way through the gel, then stained with SimplyBlue^TM^ SafeStain. The sample lane was cut into 12 equal slices, then into 1 mm cubes and destained using 50 mM ammonium bicarbonate (Ambic) containing 50% acetonitrile (ACN, v/v) at 37°C in an Eppendorf ThermoMixer Compact at 900 rpm. Samples were dehydrated (ACN, 5 min) and dried via Speedvac rotary evaporation. Peptides were reduced in 10 mM dithiothreitol (56°C, 1 h), alkylated in 55 mM iodoacetamide (RT, 30 min), washed in 100 mM Ambic (15 min) then repeated in 50 mM Ambic with 50% ACN. Samples were dehydrated in ACN, followed by Speedvac evaporation. Peptides were cleaved with 3 μg trypsin per sample lane in 40 mM Ambic and 9% ACN (37°C, overnight) then extracted using ACN (30°C, 30 min), followed by 1% formic acid (FA; RT, 20 min) and then ACN again. Supernatants were dried in a Speedvac overnight and stored at −20°C until analysis.

Samples were desalted using C18 tips (Rappsilber et al., 2003). Briefly, samples were resuspended in 50 µl 0.1% FA. C18 tips were first activated with methanol and equilibrated twice with 0.1% FA; then samples were loaded, and peptides were washed with 0.1% FA. Samples were then eluted with 80% ACN and 0.1% FA and dried in the speed vac. Finally, they were reconstituted in 0.1% FA. Peptides were then fractionated on a QExactive HF plus column coupled to a nanoHPLC Ultimate 3000 using a pre-concentration onto a nano-column configuration. An Acclaim PepMap 100 (75 µm, 2 cm) was used to do the pre-concentration and an Acclaim PepMap rapid separation liquid chromatography (RSLC; 75 µm, 15 cm) was used for peptide separation. Total run time was 118 min with a gradient from 4% to 40% buffer B (80% ACN and 0.1% FA), in the following steps: 2% for 8 min, 2% to 40% in 80 min, 90% for 10 min and an equilibration step with 2% for 20 min. The MS was operated in a data-dependent manner using a loop count of 12. MS1 was acquired in a scan range from 375–1500 *m*/*z*, with a resolution of 120000, an automatic gain control (AGC) target of 3×10^6^ and a maximum injection time (IT) of 120 ms. Tandem MS spectra were acquired at a resolution of 17,500, an AGC target of 10^5^ and maximum IT time of 60 ms. Dynamic exclusion was set to 20 s and ion with charge states of +1 and greater than +6 were excluded. Searches were performed using MaxQuant, version 1.6.17.0, against the human database. Carbamidomethylation of cysteines (+57 Da) was used as a fixed modification and oxidation of methionine (+16 Da) and acetylation of the N-terminal (+42 Da) as variable modifications. Multiplicity was set to three and the labels were as follow: medium labels, Arg6 and Lys4; heavy labels, Arg10 and Lys8.

### Microtubule analysis

The Mitochondrial Network Analysis (MiNa) plugin for Fiji (Valente et al., 2017) was utilised to characterise the interconnectivity of the MT network. The corrected total cell fluorescence (CTCF) of the MT network was analysed using Fiji and calculated using the following equation: CTCF=integrated density – (area of selected cell×mean fluorescence background). The MT network density was analysed using Fiji to calculate the percentage of empty space by applying a threshold value to the images based on the control sample.

### Statistical analysis

For western blot quantifications, band intensities were measured using Image Studio Software. All statistical analysis was carried out using GraphPad Prism. Error bars are mean±s.d. and significance is denoted not significant (ns)>0.05, *P≤0.05, **P≤0.01, ***P≤0.001, ****P≤0.0001).

## Supplementary Material

10.1242/joces.261522_sup1 Supplementary information

Table S1. Proteins identified by MS in control (Light labeled), Nocodazole (Medium labeled), and Taxol (Heavy labeled) treated cells after MT fraction isolation. Related to figure 2. Proteins that were identified/quantified in a single repeat were excluded.
