# Supplementary material for: Microtubule association of TRIM3 revealed by differential extraction proteomics
Source: J Cell Sci. 2024 Jan 31;137(2):jcs261522. doi: 10.1242/jcs.261522 (PMC10917062; doi:10.1242/jcs.261522)
Supplement: Supplementary information [file joces-137-261522-s1.pdf]

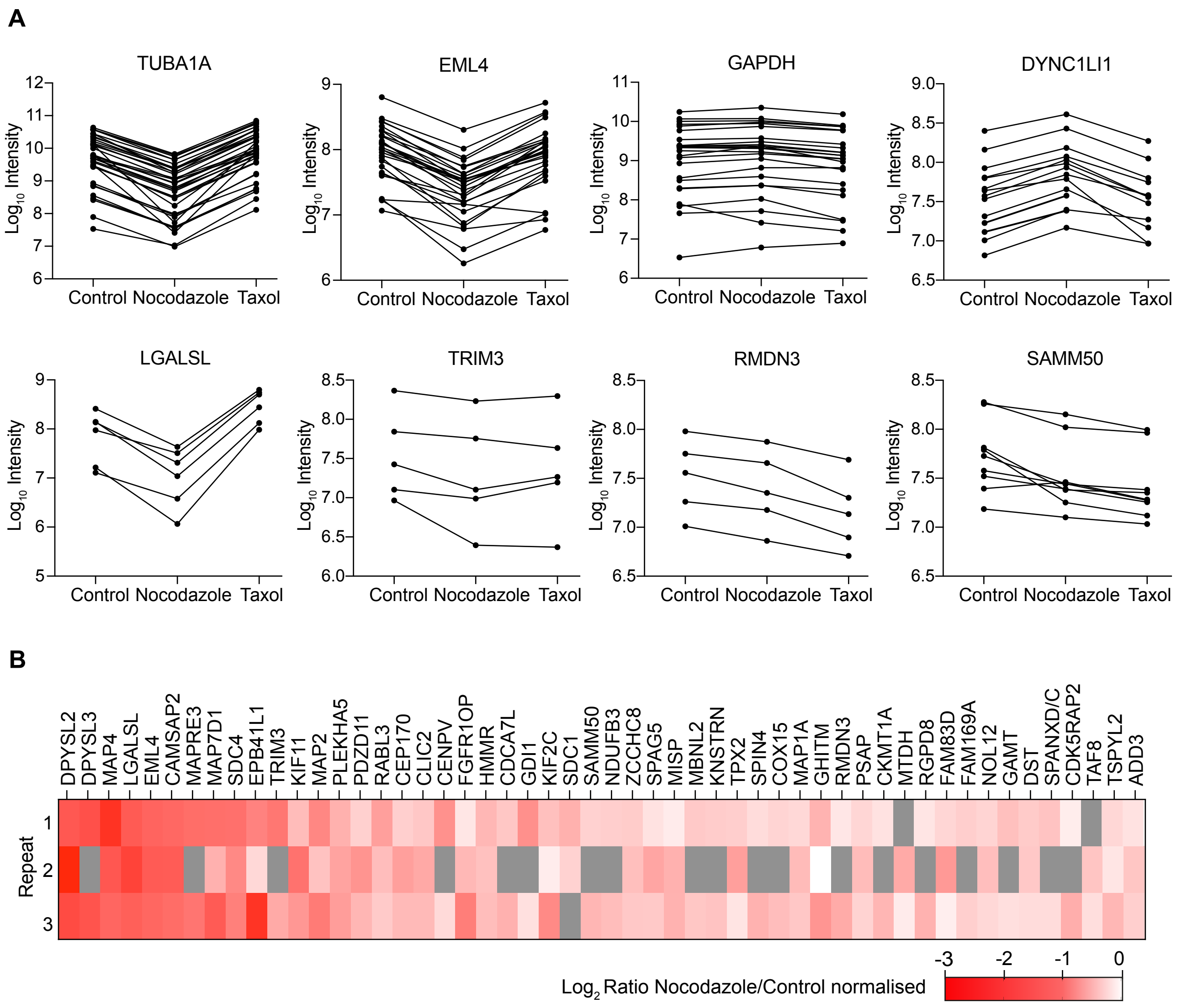

Fig. S1. Mass spectrometry results

A. Log_{10} of intensity values across the Control (DMSO only, light), Nocodazole (medium) and Taxol (heavy) conditions for each unique peptide identified for the indicated proteins.

B. Heat map showing the log_2 Nocodazole/Control normalised ratios of the proteins de-enriched in the Nocodazole fraction beyond the cut-off defined in Figure 2A (excluding Tubulin). Grey boxes indicate that a protein was not seen in that repeat.

Journal of Cell Science • Supplementary information

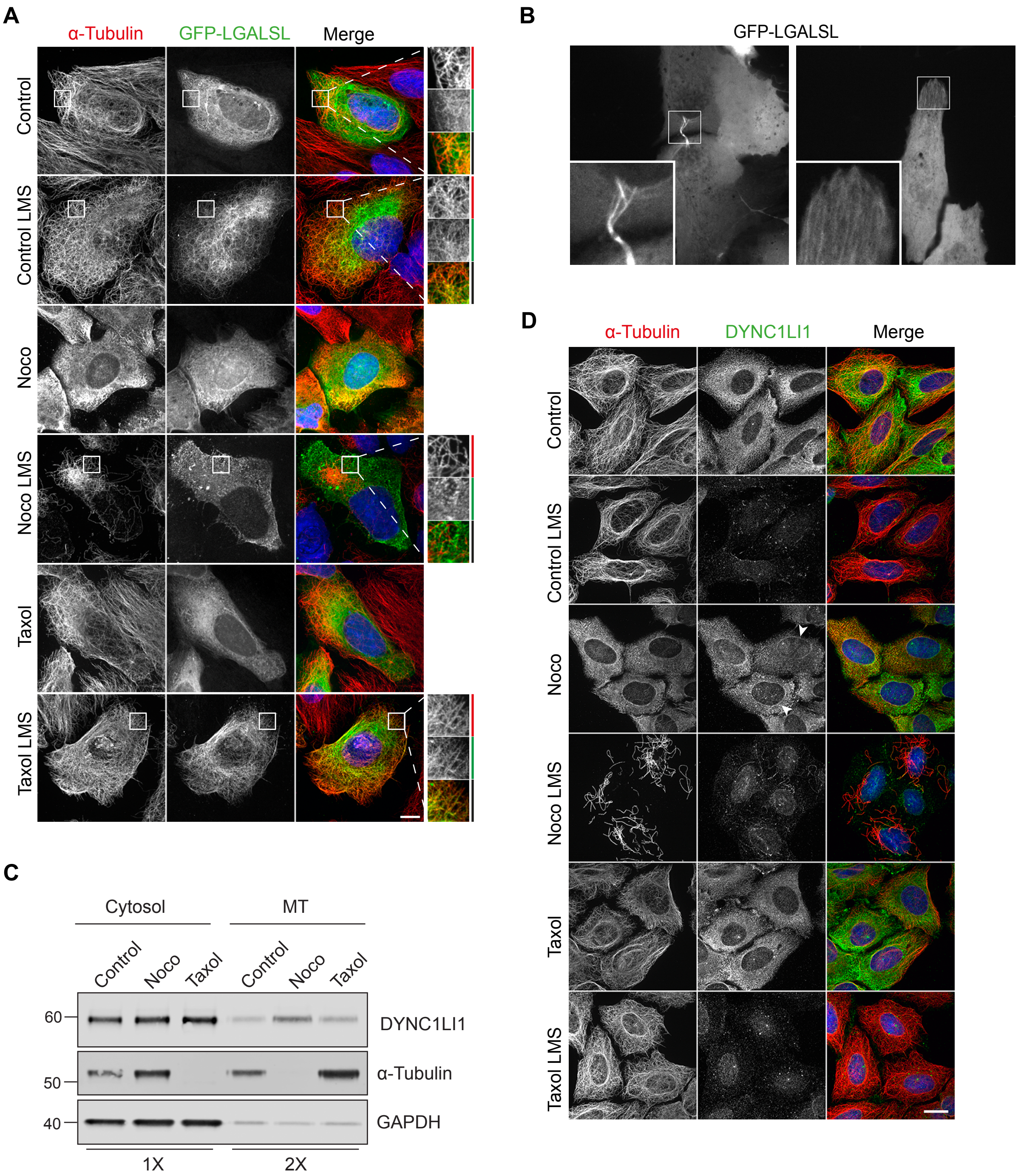

Journal of Cell Science • Supplementary information

Fig. S2. Analysis of LGALSL and dynein

A: U2OS cells were transfected with GFP-LGALSL for 21 h. Cells were then treated with 6 µM Nocodazole (Noco, 1 h) or 6μM Taxol (30 min) alongside a vehicle (DMSO) treated control sample. Cells were either fixed immediately or treated with LMS buffer (5 min on ice) prior to fixation with methanol and staining for α-Tubulin. Visualisation was performed with a 3i spinning disk confocal microscope with a Plan-Apochromat 63x/1.4NA Oil Objective M27. Scale bar: 10 µm.

B: U2OS cells were transfected with GFP-LGALSL plasmid DNA for 21 h. Images were acquired from live cells using a 3i spinning disc confocal microscope with a Plan-Apochromat 63x/1.4NA Oil Objective M27. Scale bar: 10 µm.

C: U2OS cells were treated with either 6 µM Nocodazole for 1 h or Taxol for 30 min alongside a DMSO-only treated control sample. Lysis and microtubule stabilisation buffer (LMS) was then added (5 min, 4 °C) to remove cytosolic proteins (Cytosol). Microtubules were then collected using 8 M urea lysis buffer (MT). Samples were analysed by western blot as indicated, loading twice as much MT-fraction as Cytosol.

D: U2OS cells were treated with either 6µM Nocodazole for 1 h or Taxol for 30 min alongside a DMSO control. Cells were either fixed immediately or treated with LMS (5 min on ice) prior tofixation with methanol and staining for DYNC1LI1 (green), Tubulin (red) and DAPI (blue). Visualisation was performed with a 3i spinning disk confocal microscope with a Plan-Apochromat 63x/1.4NA Oil Objective M27. Scale bar: 10 µm. White arrowheads indicate the accumulation of DYNC1LI1 at the nuclear envelope.

Journal of Cell Science • Supplementary information

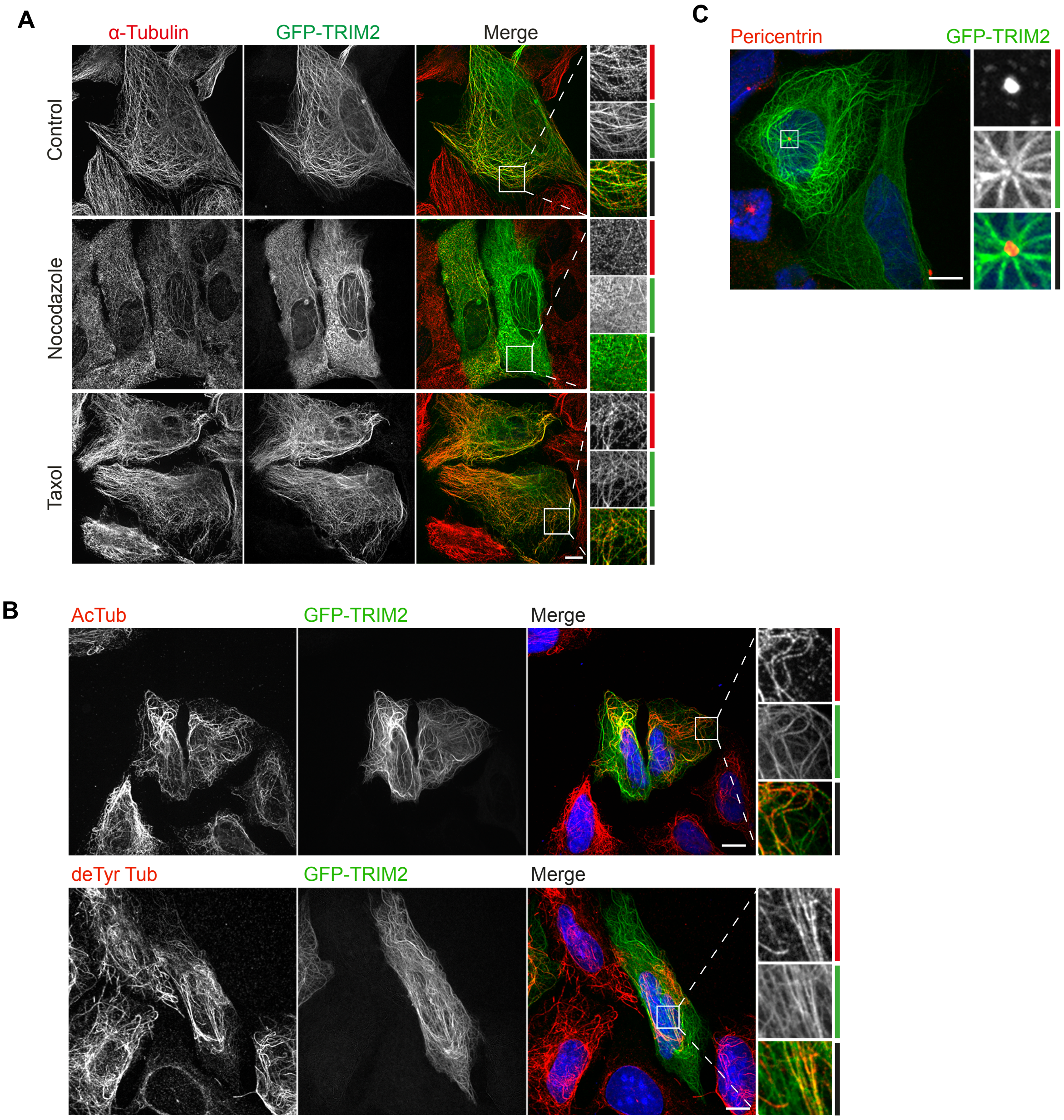

Fig. S3. TRIM2 localisation to microtubules

U2OS cells were transfected with GFP-TRIM2 for 21 h. All immunofluorescence experiments were fixed in ice-cold methanol. Visualisation was performed with a 3i spinning disk confocal microscope with a Plan-Apochromat 63x/1.4NA Oil Objective M27. Scale bar: 10 µm.

A: Transfected U2OS cells were treated with vehicle (DMSO) (1 h, control), Nocodazole (6 μM, 1 h) or Taxol (6 μM, 30 min) before fixation and staining for α-Tubulin (red).

B: Transfected U2OS cells were fixed and stained for Pericentrin (red) and DAPI (blue).

C: Transfected U2OS cells were fixed and stained for Pericentrin (red) and with DAPI (blue).

Journal of Cell Science • Supplementary information

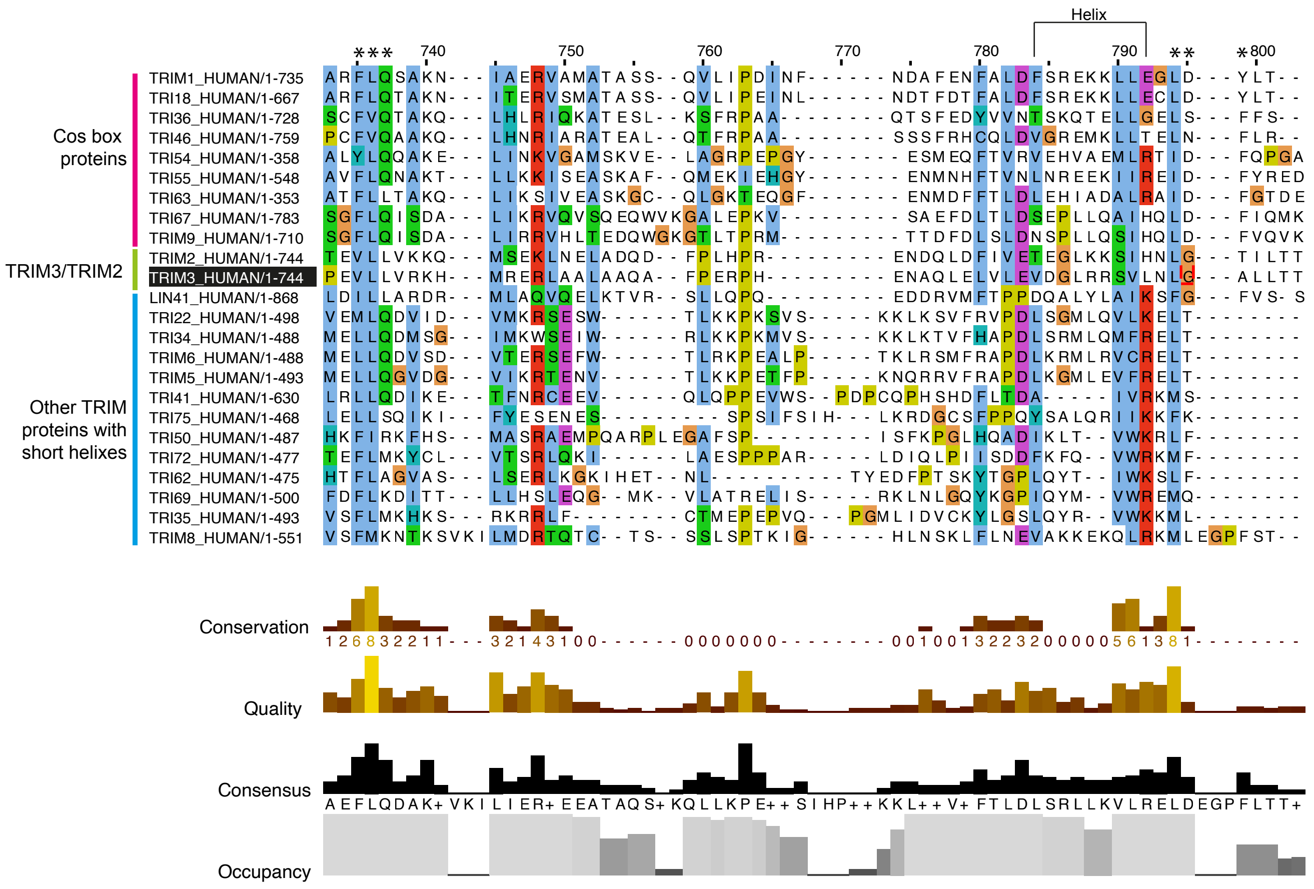

Fig. S4. TRIM3 alignment

Alignment of TRIM3 compared to the COS-box containing TRIM proteins and other TRIMs which contain a similar helical structure. The COS-box sequence was compared across all selected proteins using MAFFT alignment using L-INS-I iterative refinement. Sequences were viewed and the figure created in Jalview. The helix and conserved COS-box amino acids are indicated (*).

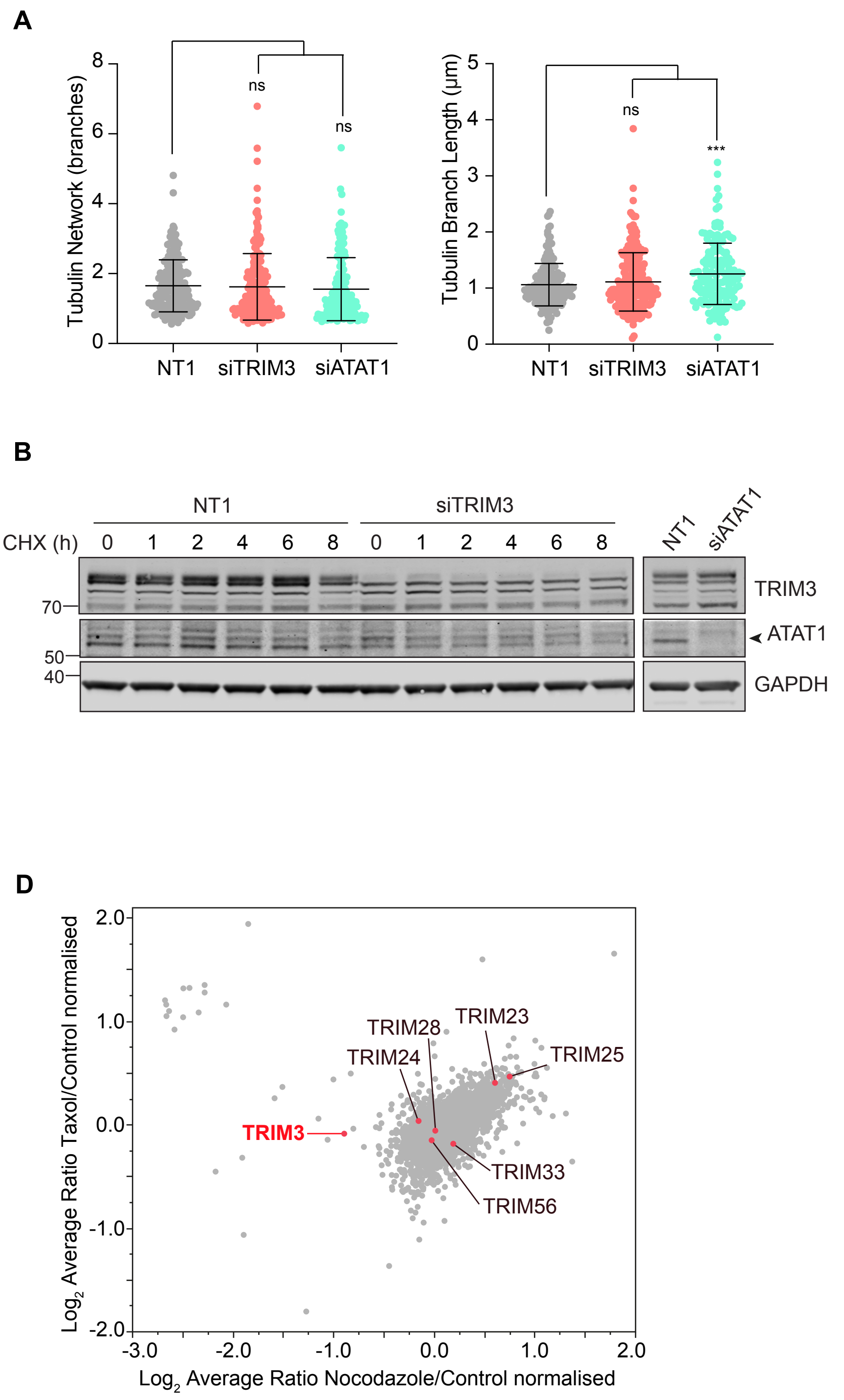

Journal of Cell Science • Supplementary information

Fig. S5. TRIM3 does not alter the stability of ATAT1

A. U2OS cells were treated as described in Figure 6C. Quantification using MiNa analysis shows the mean branch network and branch length of the whole microtubule network using the β-Tubulin staining. Cell numbers analysed: siNT1 (190), siTRIM3 (207), siATAT1 (167). Mean and SD are shown. Significance was determined using a one-way ANOVA, with Tukey's multiple comparison test.

B. U2OS cells were transfected with siRNA targeting TRIM3 for 72 h. Cells were then treated with 100 μg/ml cycloheximide (CHX) for the indicated time points before lysis in RIPA buffer. ATAT1 expression levels were analysed. In parallel, cells were treated with ATAT1 siRNA to help identify the correct band representing ATAT1 (indicated by the arrowhead). Representative western blot.

C. Quantitation of data shown in B. Mean and standard deviation of 3 independent experiments in shown.

D. Graph shows the same dataset as in 2B with the positions of all TRIM proteins identified in the dataset indicated in red.

R = rainbow marker
BR = broad range marker

Figure 1C

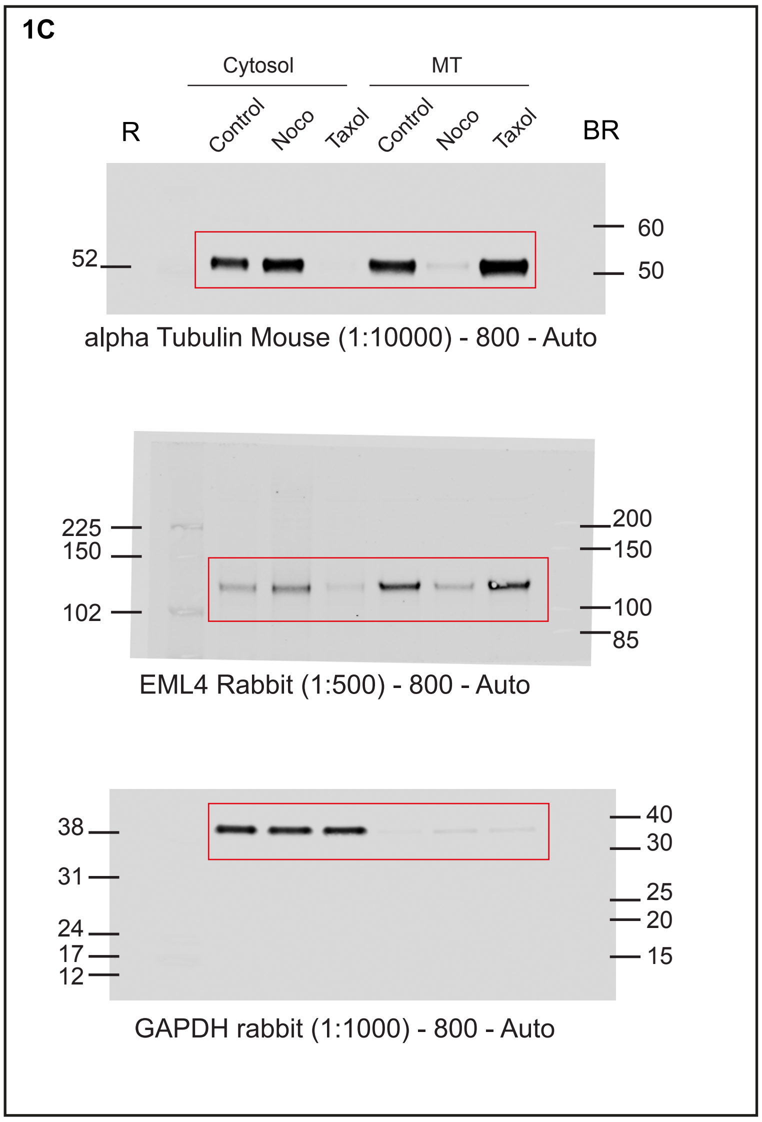

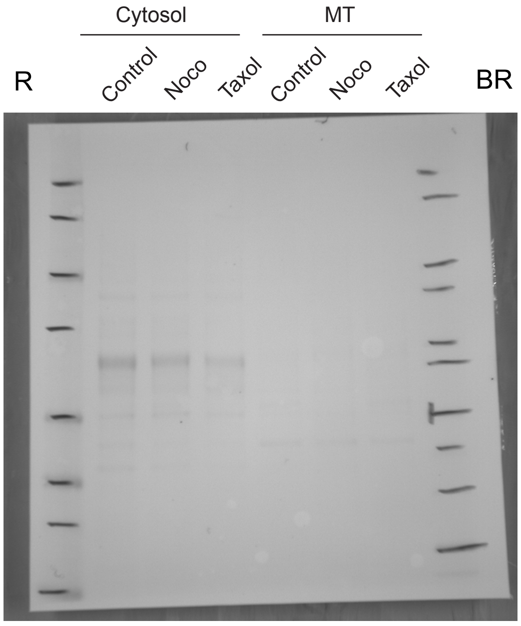

Ponceau stain

Journal of Cell Science • Supplementary information

Figure 3A

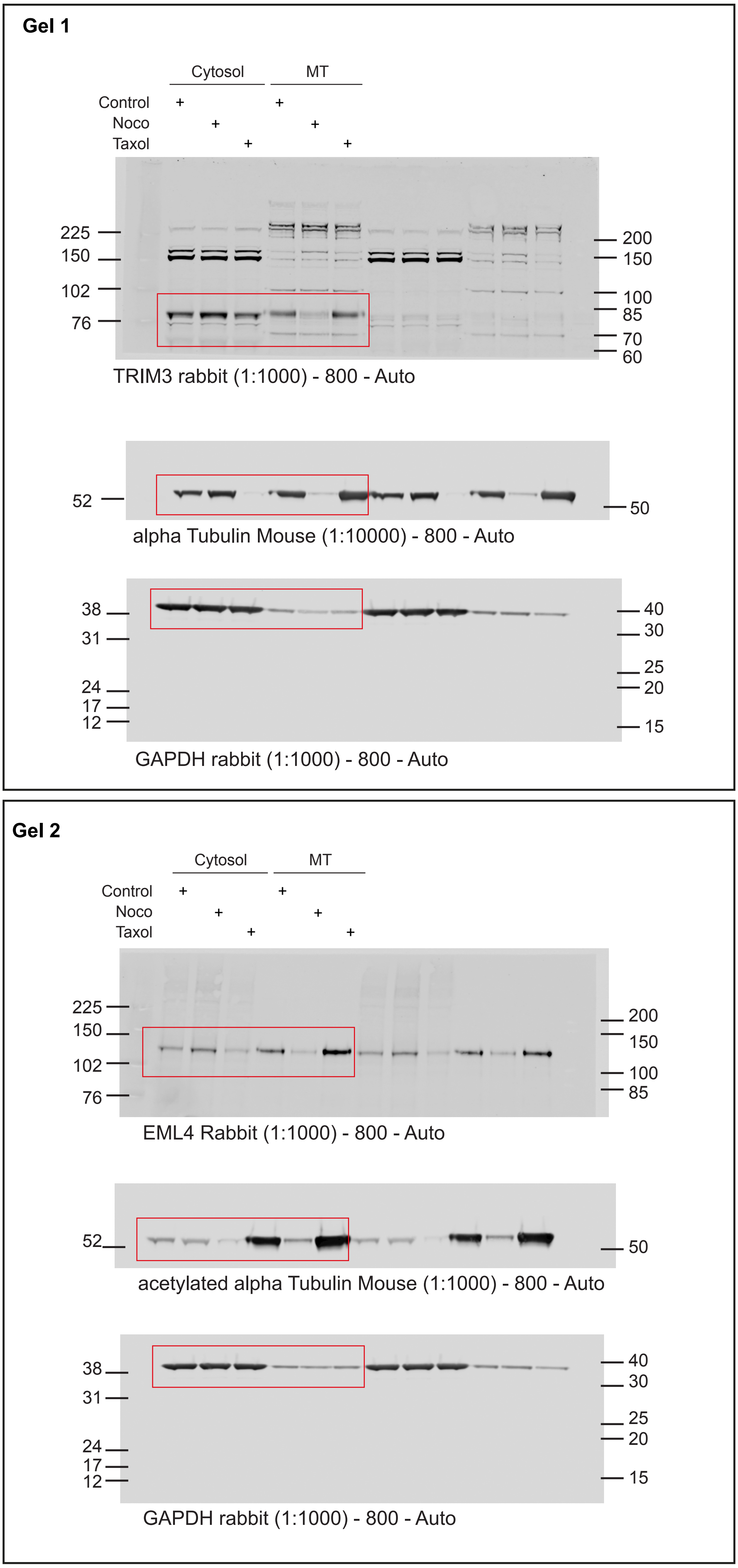

Journal of Cell Science • Supplementary information

Figure 5A

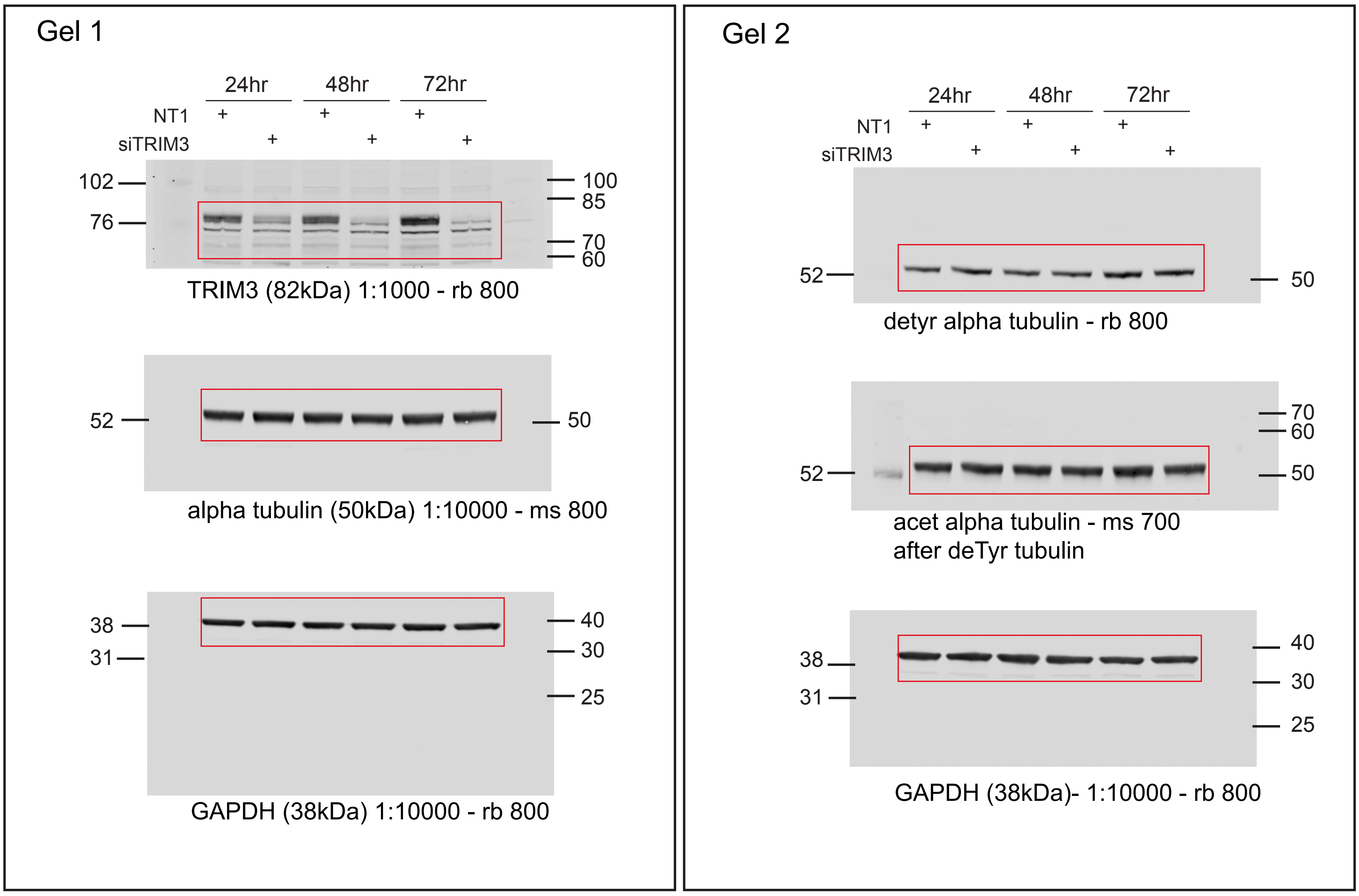
Gel 1
24hr
48hr
72hr
NT1 + + +
siTRIM3 + + +
102
76
100
85
70
60
TRIM3 (82kDa) 1:1000 - rb 800
52
50
alpha tubulin (50kDa) 1:10000 - ms 800
38
31
40
30
25
GAPDH (38kDa) 1:10000 - rb 800
Gel 2
24hr
48hr
72hr
NT1 + + +
siTRIM3 + + +
52
50
detyr alpha tubulin - rb 800
70
60
52
50
acet alpha tubulin - ms 700
after deTyr tubulin
38
31
40
30
25
GAPDH (38kDa)- 1:10000 - rb 800

Figure 6A

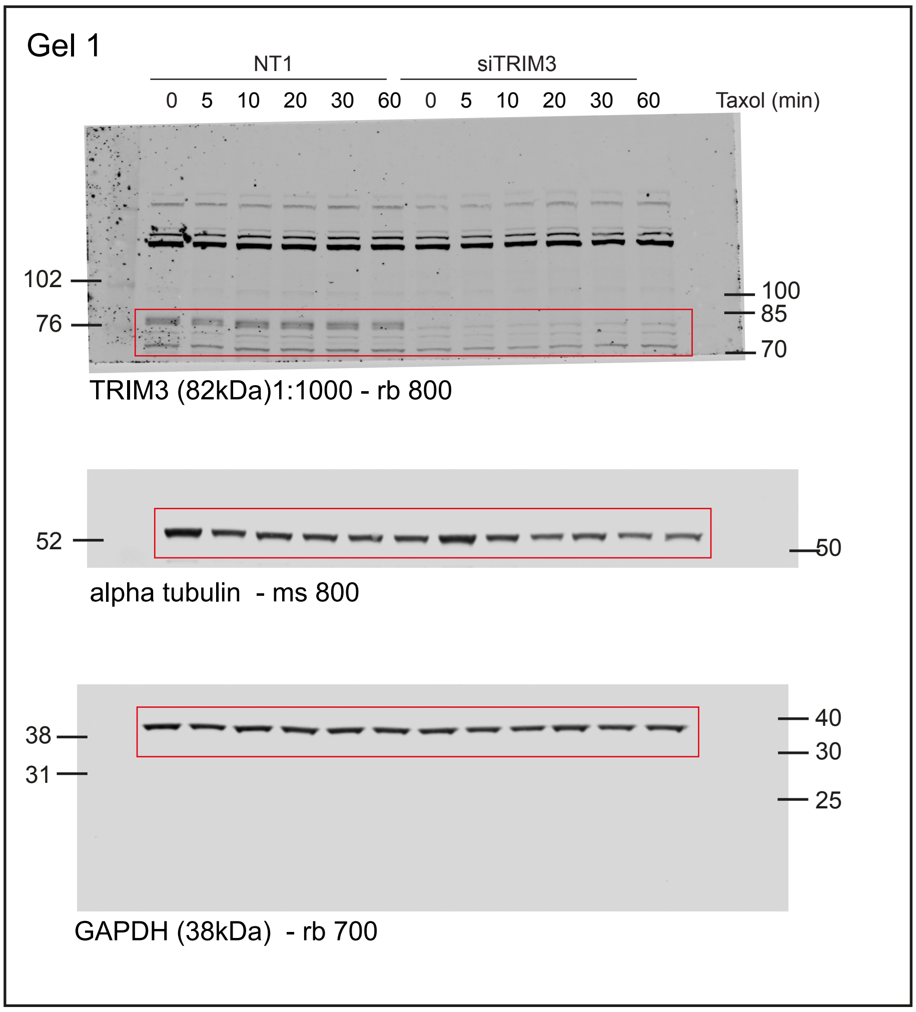

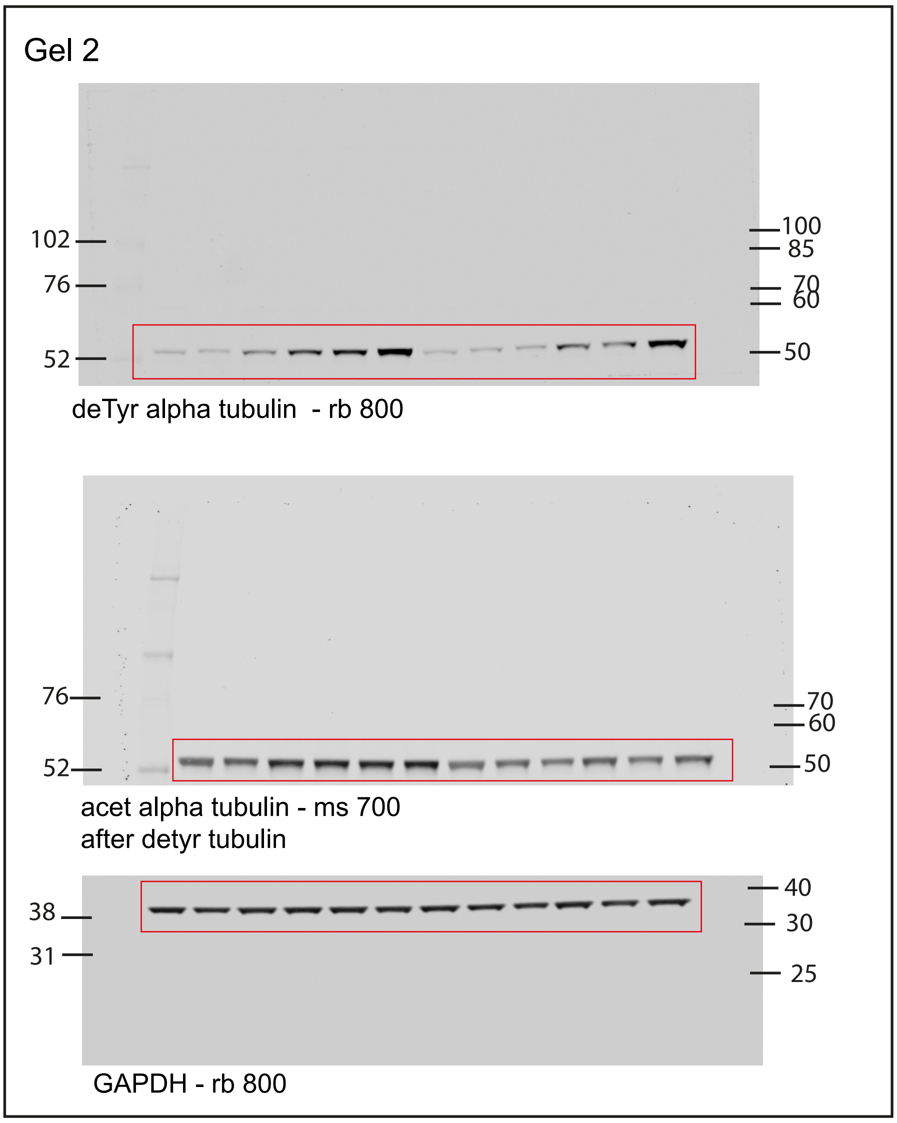

Journal of Cell Science • Supplementary information

Figure 6D

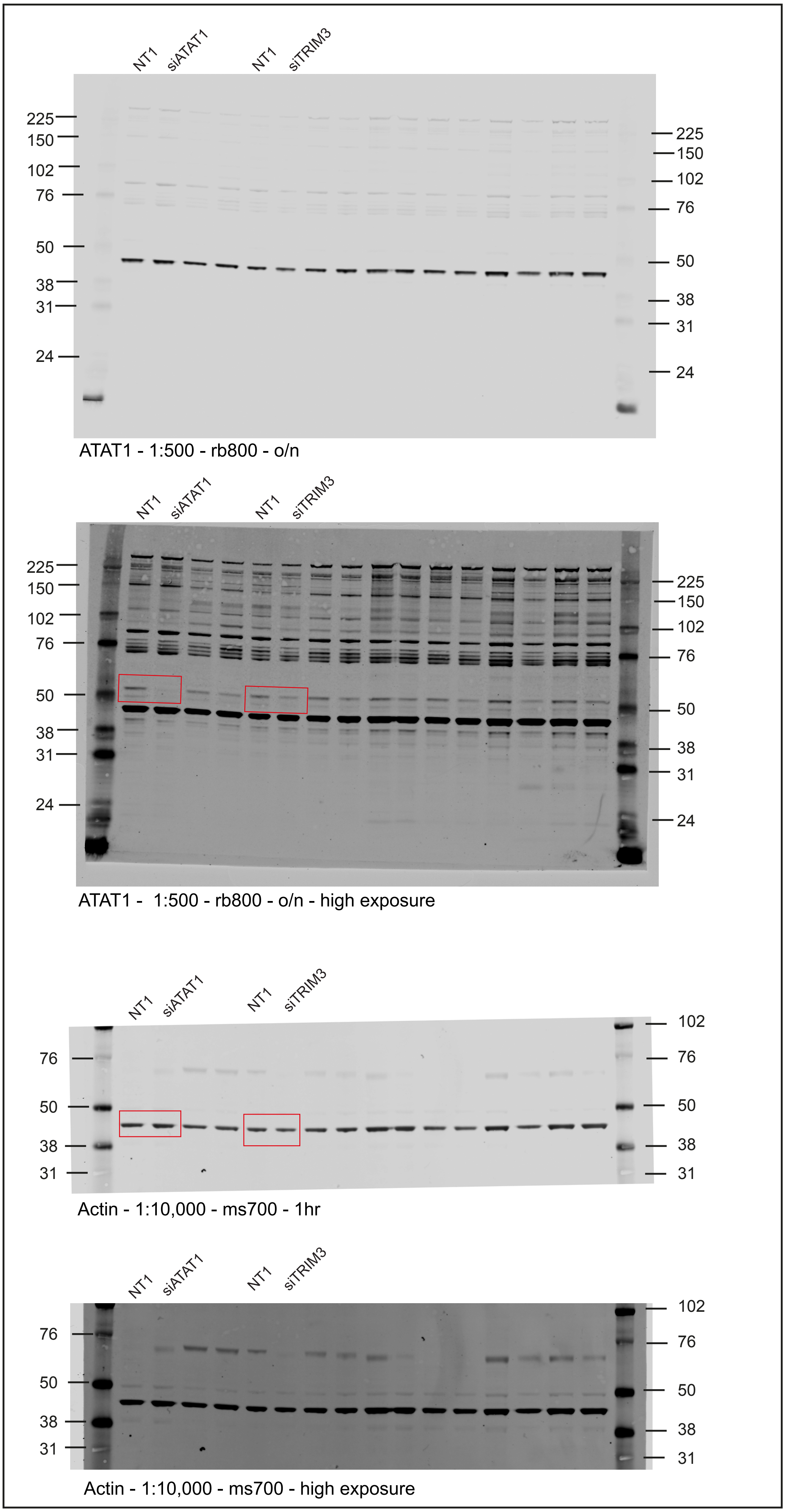

Journal of Cell Science • Supplementary information

Figure S2C

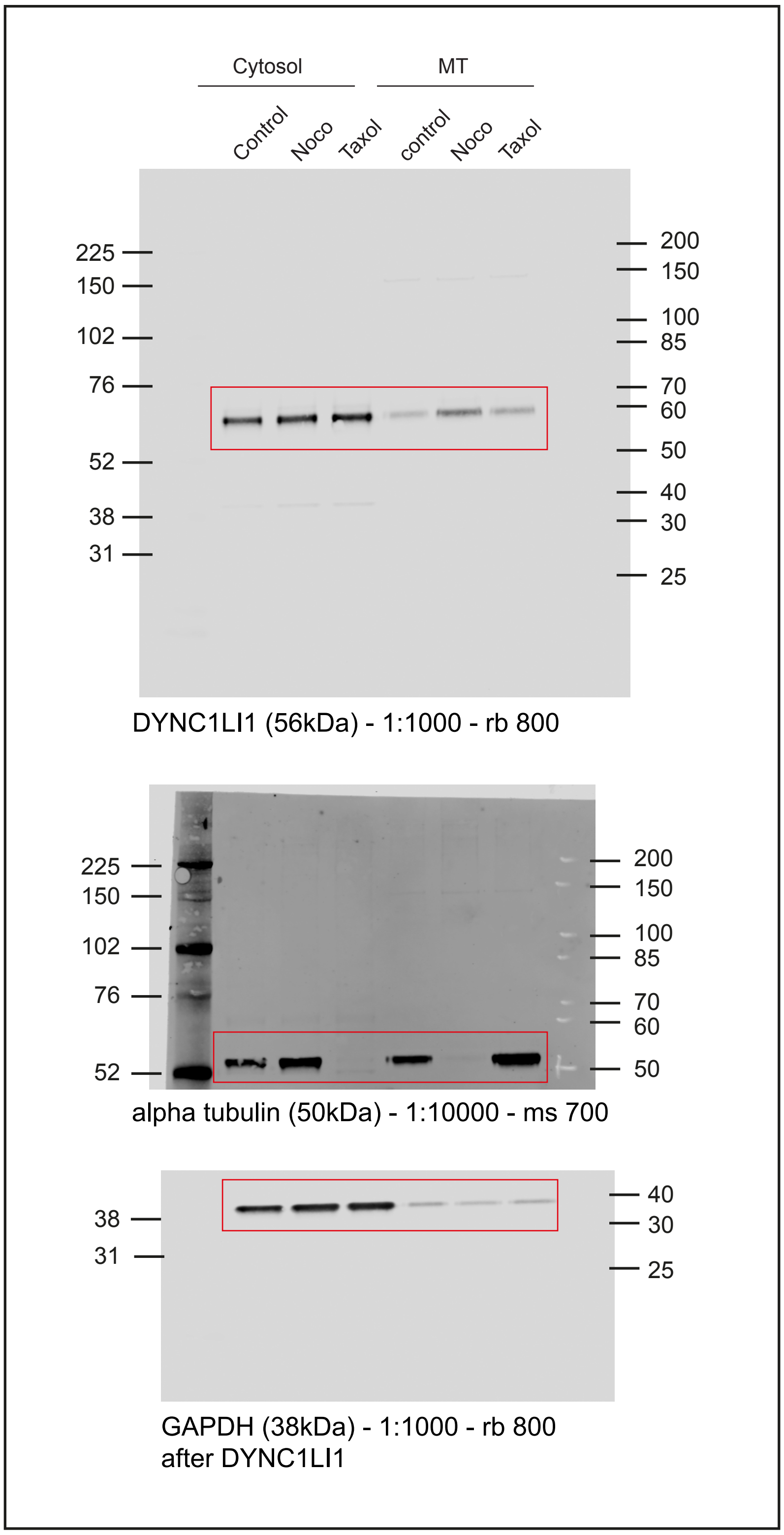

Journal of Cell Science • Supplementary information

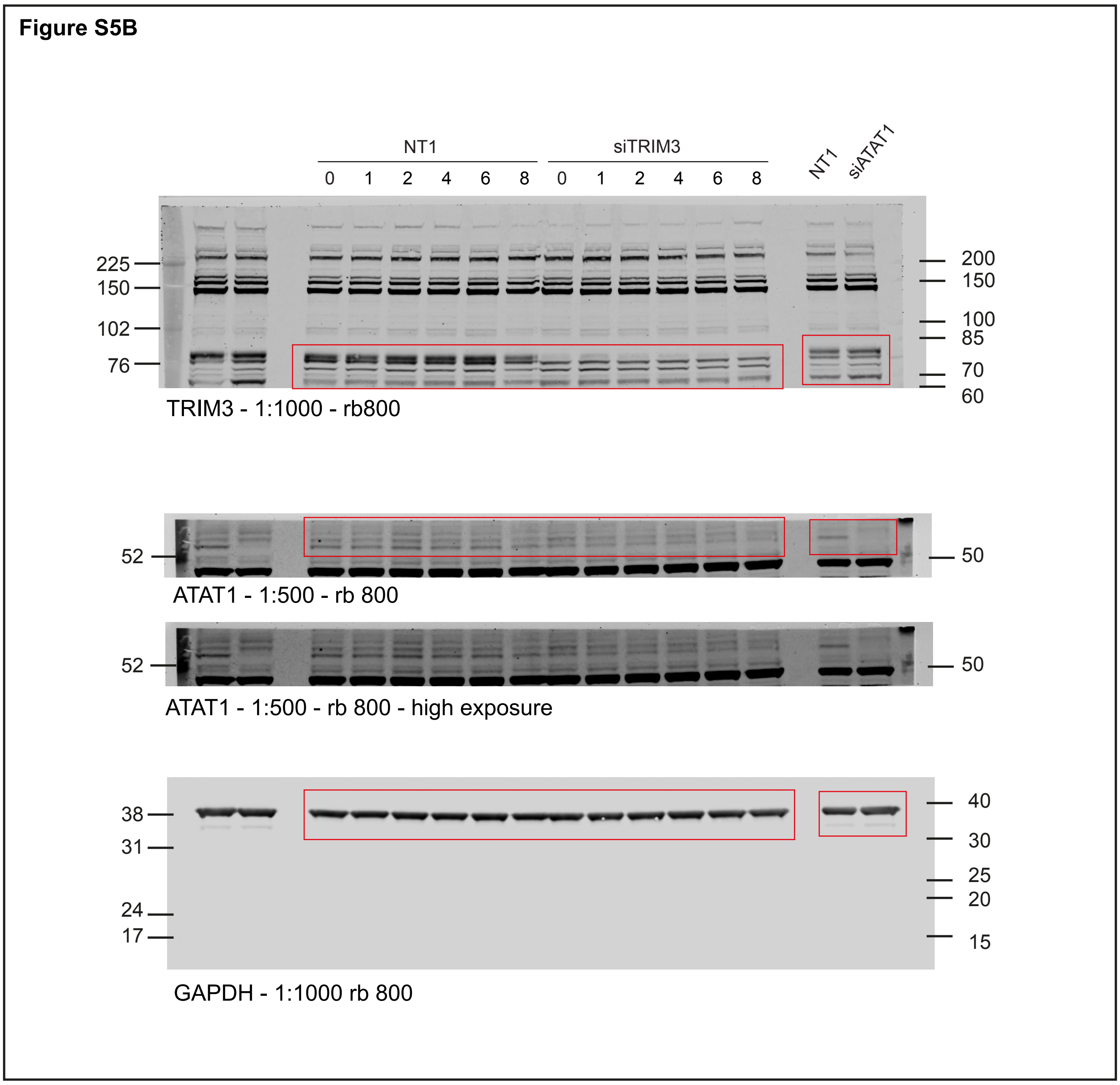

Fig. S6. Blot Transparency

Journal of Cell Science • Supplementary information

Table S1. Proteins identified by MS in control (Light labeled), Nocodazole (Medium labeled), and Taxol (Heavy labeled) treated cells after MT fraction isolation. Related to figure 2. Proteins that were identified/quantified in a single repeat were excluded.

Available for download at
https://journals.biologists.com/jcs/article-lookup/doi/10.1242/jcs.261522#supplementary-data

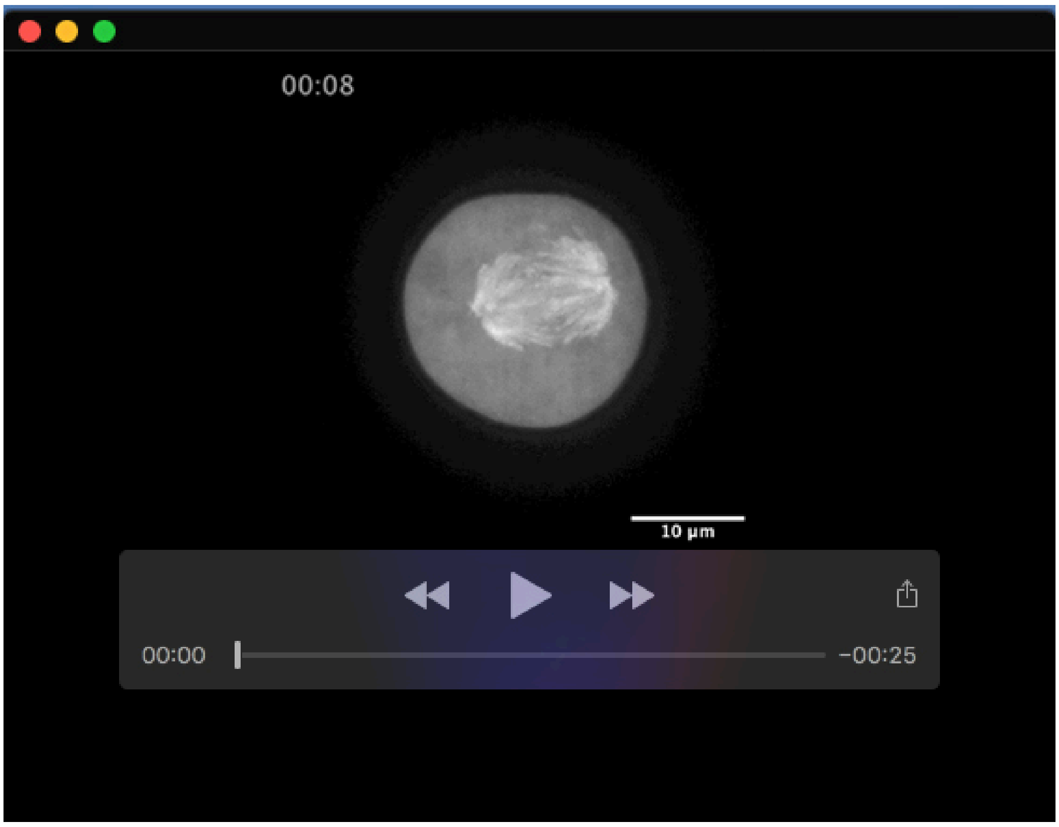

Movie 1. Movie to accompany Figure 3E. GFP-mTRIM3 transfected cells were synchronised using thymidine and Nocodazole to arrest cells at prometaphase. Nocodazole was washed out and cells placed in fresh media before Z-stacks were acquired every minute.
